# Synaptic organisation and behaviour-dependent activity of mGluR8a-innervated GABAergic trilaminar cells projecting from the hippocampus to the subiculum

**DOI:** 10.1007/s00429-020-02029-2

**Published:** 2020-02-03

**Authors:** Linda Katona, Katja Hartwich, Ryohei Tomioka, Jozsef Somogyi, J. David B. Roberts, Kristina Wagner, Abhilasha Joshi, Thomas Klausberger, Kathleen S. Rockland, Peter Somogyi

**Affiliations:** 1 Department of Pharmacology, University of Oxford, Mansfield Road, Oxford, OX1 3QT UK; 2 Laboratory for Cortical Organization and Systematics, RIKEN Brain Science Institute, Wako, Saitama 351-0198 Japan; 3 Center for Brain Research, Division of Cognitive Neurobiology, Medical University of Vienna, 1090 Vienna, Austria; 4 Present Address: Department of Morphological Neural Science, Graduate School of Medical Sciences, Kumamoto University, Kumamoto, Japan; 5 Present Address: Department of Physiology, Kavli Institute for Fundamental Neuroscience, University of California, San Francisco, CA USA; 6 Present Address: Department of Anatomy and Neurobiology, Boston University School of Medicine, 72 East Concord St., Boston, MA 02118 USA

**Keywords:** mGluR8a, Trilaminar cell, Inhibition, Burst firing, Muscarinic, Movement, Sleep, Theta, Hippocampus, Medial septum

## Abstract

In the hippocampal CA1 area, the GABAergic trilaminar cells have their axon distributed locally in three layers and also innervate the subiculum. Trilaminar cells have a high level of somato-dendritic muscarinic M2 acetylcholine receptor, lack somatostatin expression and their presynaptic inputs are enriched in mGluR8a. But the origin of their inputs and their behaviour-dependent activity remain to be characterised. Here we demonstrate that (1) GABAergic neurons with the molecular features of trilaminar cells are present in CA1 and CA3 in both rats and mice. (2) Trilaminar cells receive mGluR8a-enriched GABAergic inputs, e.g. from the medial septum, which are probably susceptible to hetero-synaptic modulation of neurotransmitter release by group III mGluRs. (3) An electron microscopic analysis identifies trilaminar cell output synapses with specialised postsynaptic densities and a strong bias towards interneurons as targets, including parvalbumin-expressing cells in the CA1 area. (4) Recordings in freely moving rats revealed the network state-dependent segregation of trilaminar cell activity, with reduced firing during movement, but substantial increase in activity with prolonged burst firing (> 200 Hz) during slow wave sleep. We predict that the behaviour-dependent temporal dynamics of trilaminar cell firing are regulated by their specialised inhibitory inputs. Trilaminar cells might support glutamatergic principal cells by disinhibition and mediate the binding of neuronal assemblies between the hippocampus and the subiculum via the transient inhibition of local interneurons.

**Electronic supplementary material:**

The online version of this article (10.1007/s00429-020-02029-2) contains supplementary material, which is available to authorized users.

## Introduction

The hippocampal formation contributes to episodic memory formation (Lorente de No 1934; Manns and Eichenbaum 2006; Squire et al. 2004) served by a complex network of excitatory cortico-cortical associative and commissural pathways including those between the entorhinal cortex, the dentate gyrus, the hippocampal areas CA1–3 and the subiculum and projections to and from subcortical areas (Amaral and Witter 1989; Colom et al. 2005; Ishizuka et al. 1990; Kohara et al. 2014; Ramon and Cajal 1893; Swanson and Cowan 1977; Wouterlood et al. 1990).

In addition to the complex network of glutamatergic pathways, an increasing number of long-range GABAergic projections have been reported, involving diverse neuronal types and synaptic mechanisms (Alonso and Köhler 1982; Basu et al. 2016; Ceranik et al. 1997; Christenson Wick et al. 2019; Eyre and Bartos 2019; Ferraguti et al. 2005; Francavilla et al. 2018; Freund 1992; Freund and Meskenaite 1992; Fuentealba et al. 2008; Jinno et al. 2007; Katona et al. 2017; Luo et al. 2019; Melzer et al. 2012; Miyashita and Rockland 2007; Ribak et al. 1986; Sik et al. 1995, 1994; Szőnyi et al. 2019; Yamawaki et al. 2019; Yuan et al. 2017). By promoting short-latency synchrony across distant cortical areas, these long-range GABAergic afferents may rhythmically bind glutamatergic principal cells into co-operative networks (Basu et al. 2016; Buzsáki and Chrobak 1995; Christenson Wick et al. 2019; Francavilla et al. 2018; Katona et al. 2017; Khodagholy et al. 2017; Yamawaki et al. 2019; Young and McNaughton 2009), thereby facilitating rhythmic neuronal network activity-dependent encoding of novel information and its consolidation and retrieval as memories (Buzsáki 1989; Hasselmo and McClelland 1999). But the specific roles of many long-range GABAergic cell types and their synaptic relationships remain to be defined.

In the hippocampal CA1, several distinct long-range projecting GABAergic neuron types have been identified coordinating neuronal activity of their targets in a behavioural‐ and rhythmic state‐dependent manner. Many of these express somatostatin (SST+) and have multi-area targets in both retrohippocampal areas, e.g. subiculum, and rostral areas, e.g. the medial septum (Christenson Wick et al. 2019; Fuentealba et al. 2008; Jinno et al. 2007; Katona et al. 2017; Miyashita and Rockland 2007). Locally, these SST+ neurons contribute to the dendritic inhibition of pyramidal cells (Jinno et al. 2007; Katona et al. 2017; Melzer et al. 2012).

A somatostatin immunonegative (SST−) GABAergic and subiculum-projecting neuron was reported in the rat—the trilaminar cell—with a number of distinctive features (Ferraguti et al. 2005; Sik et al. 1995). (1) Located in stratum oriens, the cell body and horizontally oriented dendrites express a high level of muscarinic acetylcholine receptor 2 (M2+) and receive inputs from terminals with a very high level of presynaptic metabotropic glutamate receptor 8a (mGluR8a+) (Ferraguti et al. 2005). (2) The axons projecting to subiculum are myelinated and the local axon collaterals innervate three hippocampal layers, strata oriens, pyramidale and radiatum (Ferraguti et al. 2005; Sik et al. 1995). Electron microscopic investigations have determined that at least 50% of the postsynaptic targets in the CA1 area are interneurons (Ferraguti et al. 2005). (3) In urethane-anaesthetised rats, trilaminar cells exhibit high-frequency (> 200 Hz) bursts of action potentials during sharp wave-ripple oscillations (SWRs) in contrast to their average low-frequency firing during theta oscillations (Ferraguti et al. 2005; Sik et al. 1995). It is not known if the reported activity pattern was trilaminar cell specific or a consequence of urethane anaesthesia (Ferraguti et al. 2005). In addition, information is lacking on the neurotransmitter phenotype and origin of the mGluR8a+ presynaptic terminals innervating trilaminar cells.

Recently, a vasoactive intestinal polypeptide-expressing (VIP+) GABAergic neuron type has been defined in mice also projecting to the subiculum (VIP-LRP; Francavilla et al. 2018) and with a transcriptomic profile sharing some genes with other VIP+ or long-range projecting GABAergic neurons (Harris et al. 2018; Luo et al. 2019; Paul et al. 2017). Like trilaminar cells, these neurons express high levels of somato-dendritic M2, receive mGluR8a+ inputs, preferentially make GABAergic synapses with different classes of GABAergic interneurons locally, and are only weakly active during theta oscillations associated with locomotion (Francavilla et al. 2018; Luo et al. 2019). Different from trilaminar cells, these VIP-LRP neurons have locally terminating axons innervating mostly the CA1 stratum oriens/alveus, probably promoting the synchronisation of pyramidal cell assemblies via disinhibition (Francavilla et al. 2018).

To elucidate how trilaminar cells support the functional coupling between the hippocampus and subiculum, we carried out a more in-depth analysis of these neurons in the rat, and also searched for similar neurons in the mouse, for a comparison with VIP-LRP cells. Here we determine the input–output synaptic connectivity and drug-free activity of trilaminar cells using three approaches: (1) characterisation of the boutons innervating M2+ trilaminar cells by confocal microscopy-based immunolocalisation and anterograde tracing in rat and mouse; (2) using serial section electron microscopy in combination with retrograde enhanced green fluorescent protein (EGFP) virus labelling and single-cell juxtacellular neurobiotin labelling, we quantify the postsynaptic target distribution locally in the CA1 of M2+ and mGluR8a-decorated trilaminar cells in rat; (3) we report extracellular single cell recordings using glass electrodes in freely moving rats (Katona et al. 2017; Lapray et al. 2012) targeting GABAergic neurons in stratum oriens exhibiting high-frequency (> 200 Hz) bursts of action potentials.

## Materials and methods

### Experimental animals

Animal experiments were performed according to the UK Animals (Scientific Procedure) Act 1986 and under the approval of the UK Home Office and of the Animal Care and Use Committee of the University of Oxford; and according to the NIH Guidelines for the Care and Use of Laboratory Animals (NIH Publication No. 80-23; revised 1996) approved by the Experimental Animal Committee of the RIKEN Institute. Data reported are from 62 male adult Sprague–Dawley rats (200–565 g, CD, Charles River Laboratories), 1 female and 5 male adult C57BL6/J mice (4–6 months; Charles River Laboratories), 5 male adult VGAT^Cre^ mice (5–7 months; 3 homozygote and 2 heterozygote; The Jackson Laboratory, Bar Harbor; stock #016962; kind donation from Prof. William Wisden) and 4 male adult PV^Cre^ heterozygote mice (8–14 months; The Jackson Laboratory; stock #008069). Animals were housed in groups of 2–4 per cage (19–21 °C; 55% humidity; 12/12-h light–dark cycle) with ad libitum access to food pellets and water.

### Anterograde tracing

#### Iontophoretic injections

*Phaseolus vulgaris* leucoagglutinin (PHAL; Vector Laboratories; 2.5% in 0.1 M PB solution) was iontophoretically injected (Gerfen and Sawchenko 1984) using a glass pipette with tip diameter of 12–18 μm into the medial septum of rats and mice (stereotaxic coordinates relative to Bregma: in rat, 0.6 mm anterior, 1.4 mm lateral and 5 mm, 5.5 mm and 6 mm ventral with 15° angle; in mouse, 0.85 mm anterior, 0 mm lateral and 3.6 mm ventral with 0° angle). Positive current pulses of 5 μA were applied every 7 s for 15–30 min. To minimise tissue damage and dorsal diffusion, the electrode was lowered into place 15 min before the start and was retracted 5–10 min after the end of stimulation. Three to seven days after injections, animals were perfusion fixed (4% PFA) and the brains were processed (see below).

#### Virus injections

Anterograde Cre-dependent rAAV2-CAG-FLEX-ArchT-GFP (UNC Vector Core, 2.0 × 10^12^ titer; *n* = 5 VGAT^Cre^ mice and 3 PV^Cre^ mice) or rAAV8/PAAV-hSyn-DIO-EGFP (UNC Vector Core; 8 × 10^12^ titer; *n* = 1 PV^Cre^ mouse) were pressure injected (400 nl/mouse) using a glass pipette (5 μl; Harvard Apparatus) with tip diameter of 10–15 µm into the medial septum of mice (stereotaxic coordinates relative to Bregma: 0.86 mm anterior, 0.39 mm lateral and 3.75 mm ventral with 5° angle). Pressure was applied using a 1-μl syringe at a rate of ~ 100 nl/min. To minimise tissue damage and dorsal diffusion, the electrode was lowered into place 5 min before and was retracted 10 min after the injection. After a minimum of 28 days and allowing optimal virus expression, mice were perfusion fixed (2% PFA) and the brains were processed (see below).

### Retrograde tracing using recombinant EGFP-adenovirus vector

Experimental protocols were approved by the Safety Division of the RIKEN Institute in accordance with National Institutes of Health (NIH) Guidelines for Research Involving Recombinant DNA Molecules. The adenoviral vector (Ad) is based on human adenovirus type 5, and is replication incompetent because it lacks the E1 and E3 regions of its genome and it expresses enhanced green fluorescent protein (EGFP) under the control of the neuron-specific promoter synapsin I (Syn) as described previously (Tomioka and Rockland 2006). The Ad-Syn-EGFP was purified and concentrated by double cesium step gradient centrifugation and dialyzed in 50 mM Tris–HCl (pH 8.1) containing 600 mM NaCl. The titers of viral stocks were determined by plaque assay on HEK 293 cells and adjusted to more than 1.0 × 10^12^ pfu/ml before the injection.

The Ad-Syn-EGFP retrograde virus (1.0 × 10^12^ titer; *n* = 22 CD rats) was pressure injected (0.2–0.3 µl/rat) using a glass pipette with tip diameter of 15–20 µm into the dorsal subiculum (stereotaxic coordinates from Bregma: 5.8 mm posterior; 2.0 mm lateral; 2.8 mm ventral with 0° angle). After 2 weeks, rats were perfusion fixed (4% PFA) and the brains were processed (see below).

### In vivo extracellular single cell recording and juxtacellular neurobiotin labelling in freely moving rats

#### Surgical procedures

Implantation of the head-mounted recording setup, craniotomy and duratomy were performed as previously published (Katona et al. 2017; Lapray et al. 2012). Briefly, rats were anaesthetised (IsoFlo, Abbott), placed into a stereotaxic frame (Kopf Instruments), a micro-drive holder together with a main connector supported by five stainless steel screws was attached to the skull using dental acrylic (Refobacin R, Biomet) and everything was coated with blue light-polymerised cement (Tetric EvoFlow, Ivoclar Vivadent). One of the screws was connected to recording EEG and another for reference-ground signals. After a week, either a single wire electrode (50-μm tungsten, California Fine Wire) fixed on the skull or movable by a miniature drive (Haiss et al. 2010) or a tetrode movable by a microdrive was lowered into the hippocampus or cortex. A layer of silicone (Kwik-Sil, World Precision Instruments) was used to protect the cortical surface overnight and between recordings.

#### Recordings

On recording days (1–12 days after duratomy), after short anaesthesia, a miniature preamplifier (ELC mini-preamplifier, NPI Electronic), two LEDs and an accelerometer (Supertech Instruments) were connected to the head stage. A glass electrode (10–20 MΩ) filled with neurobiotin (Vector Laboratories Ltd, 1.5 or 3% w/v in 0.5 M NaCl) was advanced using a hydraulic (Narishige) microdrive to record single cell activity and LFPs in the hippocampus. During recordings, the protective layer of silicone was replaced by paraffin wax (Sigma-Aldrich) for the ease of advancement of the glass electrode. Recordings commenced 1 h after recovery from anaesthesia in a darkened room using a recording arena (50 × 50 cm floor, 27 cm walls) to which the rats were naive on the first day. Two video cameras captured the free movements of the rats; one infra-red sensitive for behavioural analysis and another one for position tracking using the head-mounted LEDs (Position Tracking System, v14.02.08, courtesy of K. Allen). After recording a neuron, the pipette was advanced towards the cell into a juxtacellular position and labelling using neurobiotin was attempted (Pinault 1996). The cell was stimulated by 200 ms of gradually increased positive current pulses to modulate its firing using ELC-03M amplifier with active bridge compensation. When labelling was judged successful due to strong modulation of the cell’s firing, the pipette was retracted slowly and the neuron was left to recover from the entrainment. The rats were then briefly anaesthetised in the stereotaxic frame using isoflurane while the recording equipment was removed. Following a post-labelling period of up to 3 h, the animals were deeply anaesthetised using isoflurane followed by intraperitoneal injection of an overdose of Pentobarbital (JML Biopharm; 20% w/v solution; dose: 400 μl/100 g) and perfusion fixed (4% PFA; see below). If no stable neurons were found, sessions were finished by simply removing the recording devices and by covering the cortical surface with a layer of silicone between consecutive days.

#### Recording technical specifications/data acquisition

Electrophysiological signals were amplified 1000× (BF-48DGX and DPA-2FS, NPI Electronic) and were digitised at 20 kHz (Power1401 A/D board, Cambridge Electronics Design). Measurements from the glass electrode were online bandpass filtered according to three different frequency ranges (0.3 Hz–10 kHz, wide-band; 0.3–500 Hz, LFP; 0.8–5 kHz, action potentials). Signals from the EEG and hippocampal/cortical electrodes or tetrodes were bandpass filtered (0.3 Hz–10 kHz). Elimination of 50 Hz noise without phase-shift was provided by Hum Bugs (Quest Scientific Instruments). Accelerometer measurements were digitised at 20 kHz. Acquisition of all signals, except video tracking, went in parallel using Spike2 software (v7.06a, Cambridge Electronic Design, ced.co.uk).

### Behavioural state detection and electrophysiological data analyses

The detection of slow wave sleep, paradoxical sleep, quiet wakefulness and movement was carried out using previously published criteria (Katona et al. 2017; Lapray et al. 2012). Specifically, periods of movement were identified based on motion tracking and these included slight head movements together with all episodes of running without speed filtering. In comparison, motionless awake episodes were categorised as quiet wakefulness. For the recordings lacking accelerometer measurements, LED array-tracking data were imported into Spike2 for movement detection. We have defined sleep–wake transitions as intervals at the end of slow wave sleep periods when the cortical LFP or EEG measured from a screw in the skull oscillates at low delta frequencies (< 3 Hz), it is enriched in spindle oscillations (9–14 Hz) and, simultaneously, the hippocampal LFP oscillates at theta frequencies (5–12 Hz). We have also detected theta oscillatory epochs (5–12 Hz) using Spike2 and MATLAB (Wavelet Toolbox, v7.9-R2009b, MathWorks, uk.mathworks.com). Where possible, LFPs were analysed from the implanted single wire electrodes and/or tetrodes. Otherwise, the detection was done on LFPs measured by the glass electrode in stratum oriens, where theta oscillations are in phase with those measured in the pyramidal cell layer. Due to spike contamination of the LFP in spectral components above 100 Hz—as a result of the large action potential amplitude and high-frequency firing of the trilaminar cell—we were unable to detect individual SWR (130–230 Hz) events in the recorded LFP. Periods suggestive of the mechanical influence of the glass electrode on the firing of the recorded cell were excluded from further analyses.

For each cell, we have calculated average firing rates, point estimates (median ± interquartile range) for the inter-spike interval distributions, autocorrelograms and the rate of occurrence of action potential bursts in the spike trains during different behavioural and oscillatory network states using MATLAB. From the last, we have derived the burst frequency index using the formula:

$$\frac{\mathrm{b}\mathrm{u}\mathrm{r}\mathrm{s}\mathrm{t}\mathrm{F}\mathrm{r}\mathrm{q}\mathrm{M}\mathrm{O}\mathrm{V}- \mathrm{b}\mathrm{u}\mathrm{r}\mathrm{s}\mathrm{t}\mathrm{F}\mathrm{r}\mathrm{q}\mathrm{S}\mathrm{W}\mathrm{S}}{\mathrm{b}\mathrm{u}\mathrm{r}\mathrm{s}\mathrm{t}\mathrm{F}\mathrm{r}\mathrm{q}\mathrm{M}\mathrm{O}\mathrm{V}+ \mathrm{b}\mathrm{u}\mathrm{r}\mathrm{s}\mathrm{t}\mathrm{F}\mathrm{r}\mathrm{q}\mathrm{S}\mathrm{W}\mathrm{S}},$$

where burstFrqMOV and burstFrqSWS are the frequency of burst occurrence in Hz during movement and slow wave sleep, respectively.

Furthermore, we quantified the depth of theta modulation of each cell using Rayleigh’s uniformity test (Zar 1999) and we computed the preferential mean theta phase of firing using normalised vector addition.

For all methods, *p* values and confidence intervals were calculated according to *α* = 0.05 and the analyses were performed using standard functions and custom-written code in MATLAB (Statistical Toolbox). Chi-square test was used to compare between two distributions.

### Anatomical analyses

#### Tissue processing

Animals were anaesthetised with either sodium pentobarbital (50 mg/kg, i.p.) or chloral hydrate (350 mg/kg) and transcardially perfused with saline followed by either 2% or 4% paraformaldehyde (wt/vol, PFA, Sigma-Aldrich), 15% saturated picric acid (vol/vol, Sigma-Aldrich) and 0.05% glutaraldehyde (wt/vol, distilled grade, TAAB Laboratories Equipment Ltd) in 0.1 M phosphate buffer (PB) at pH 7.2. After brain removal, coronal sections were produced (70 μm nominal thickness, VT1000s vibratome, Leica Instruments). Sections were stored in 0.1 M PB with 0.05% sodium azide at 4 °C.

#### Immunohistochemistry

For immunohistochemistry, indirect primary antibody detection method was used in combination with fluorochrome-conjugated secondary antibodies as described before (Katona et al. 2017; Lasztoczi et al. 2011; Unal et al. 2015). The expression of cell type-specific molecules was tested on either virally transfected individual cells or cells recorded and labelled using neurobiotin and on control tissue. Immunoreactivity was assessed visually by comparing neighbouring cells within the same field of view. A positive signal in the cell of interest was accepted if the subcellular location (e.g. plasma membrane), pattern, and strength of the signal were as expected and similar to those in unlabelled cells. Cells were considered immunonegative for a molecule when fluorescence was undetectable in the tested part of the cell in an area where similar parts of other unlabelled cells were immunopositive. Primary antibodies were used for the following molecules at given dilutions: calbindin (CB, Swant Cat# CB 38, RRID:AB_10000340, 1:500, 1:5000); cannabinoid receptor-1 (CB1, Frontier Institute Cat# CB1-GP, RRID:AB_2571593, 1:1000 and Frontier Institute Cat# CB1-Rb, RRID:AB_2571591, 1:1000); choline acetyltransferase (ChAT, Synaptic Systems Cat# 297 013, RRID:AB_2620040, 1:1000); calretinin (CR, Swant Cat# CG1, RRID:AB_10000342, 1:1000 and Synaptic Systems Cat# 214 102, RRID:AB_2228331, 1:500); enhanced green fluorescent protein (EGFP, gifted by Prof. K. Rockland, RIKEN, rabbit, (Tomioka and Rockland 2006), 1:100, 1:2000 and guinea pig, (Tomioka and Rockland 2007), 1:1000); glutamate decarboxylase (GAD, Millipore Cat# MAB351, RRID:AB_2263126, 1:500); green fluorescent protein (GFP, Molecular Probes Cat# A-11120, RRID:AB_221568, 1:5000 and Aves Labs Cat# GFP-1020, RRID:AB_10000240, 1:1000, 1:10,000); muscarinic acetylcholine receptor M2 (Millipore Cat# MAB367, RRID:AB_94952, 1:250, 1:400, 1:1000); metabotropic glutamate receptor-7a [mGluR7a, gifted by Prof. R. Shigemoto, Division of Cerebral Structure, Nat. Inst. Physiol. Sci, Okazaki, rabbit, (Shigemoto et al. 1996; 1997), 1:500]; metabotropic glutamate receptor-8a [mGluR8a, gifted by Prof. R. Shigemoto, Division of Cerebral Structure, Nat. Inst. Physiol. Sci, Okazaki, guinea pig, (Kinoshita et al. 1996), 1:500, 1:2000 and Santa Cruz Biotechnology Cat# sc-30300, RRID:AB_2116478, 1:800, 1:1000, 1:2000 requiring 2% PFA]; Netrin-G1 (rabbit, 1:1000); Phaseolus vulgaris-leucoagglutinin (PHAL, Vector Laboratories Cat# AS-2224, RRID:AB_2315141, 1:5000 and Vector Laboratories Cat# AS2300, RRID:AB_2315142, 1:500); parvalbumin [PV, Swant Cat# 235 (from 2009), mouse, 1:5000, 1:7500 and Swant Cat# PV27, RRID:AB_2631173, 1:500 and Synaptic Systems Cat# 195 004, RRID:AB_2156476, 1:5000]; somatostatin [SM, gifted by A. Buchan, Medical Research Council Regulatory Peptide Group, Vancouver, British Columbia, mouse, (Vincent et al. 1985), 1:200 and GenWay Biotech Inc. Cat# 18-783-76392-1 ml, RRID:AB_1027453, 1:100]; vesicular acetylcholine transporter (VAchT, Millipore Cat# ABN100, RRID:AB_2630394, 1:3000); vesicular GABA transporter (VGAT, Frontier Institute Cat# VGAT-Rb, RRID:AB_2571622, 1:500 and Synaptic Systems Cat# 131 003, RRID:AB_887869, 1:500 and Frontier Institute Cat# VGAT-Go, RRID:AB_2571623, 1:500 and Synaptic Systems Cat# 131 004, RRID:AB_887873, 1:500); vesicular glutamate transporter-1 (VGLUT1, Synaptic Systems Cat# 135 311, RRID:AB_887880, 1:500); vesicular glutamate transporter-2 (VGLUT2, Synaptic Systems Cat# 135 402, RRID:AB_2187539, 1:500 and Synaptic Systems Cat# 135 404, RRID:AB_887884, 1:1000, 1:2000); vasoactive intestinal polypeptide (VIP, gifted by G. Ohning, Cure, UCLA, mouse, (Wong et al. 1996), 1:30,000, 1:50,000 and ImmunoStar Cat# 20077, RRID:AB_572270, 1:1000). Specificity information for primary antibodies is provided in Salib et al. (2019) and Viney et al. (2018). Secondary antibodies were reported in Katona et al. (2017). Neurobiotin was visualised by streptavidin-conjugated fluorochromes mixed with the solution.

#### Quantification of mGluR8+ terminals in apposition to M2+ neurons

Z-stacks of 8-bit images were taken of M2+ somata and proximal dendrites using laser scanning confocal microscopy and DIC M27 Plan-Apochromat 63×/1.4 NA or alpha Plan-Apochromat 100×/1.46 NA objectives. Assignment of antibodies and respective fluorophores was based on minimising spectral overlap between neighbouring channels and voxel size was optimised for the mGluR8a channel. Counting was carried out after applying a median filter (see below) to reduce noise. Immunolabelled boutons in apposition to M2+ trilaminar cells were counted from individual optical slices. At each level, mGluR8a+ terminals were selected, checked for co-labelling in other channels within the same and adjacent slices and scored for immunoreactivity. Using stereological principles, the tops of varicosities were counted as positive for one or both of the markers. We have sampled: (1) mGluR8a+ boutons on 35 cells from 3 C57BL/6J mice (2% PFA); (2) mGluR8a+ and GAD+ boutons on 13 M2+ cells from 1 rat (4% PFA); (3) mGluR8a+ and VGAT+ boutons on 15 M2+ cells from 3 rats (4% PFA); (4) mGluR8a+ and VGluT1+ boutons on 3 M2+ cells from 3 rats (4% PFA); (5) mGluR8a+ and VAchT+ boutons on 4 M2+ cells from 2 rats (4% PFA); (6) mGluR8a+ , GAD+ and mGluR7a+ boutons on 9 M2+ cells from 3 rats (4% PFA); (7) PHAL+ and/or mGluR8a+ boutons on 4 M2+ cells in 1 section from 1 rat (4% PFA); (8) GFP+ and VGAT+ boutons from 10 sample views in 1 section from 1 VGAT^Cre^ mouse (2% PFA); (9) GFP+ and/or mGluR8a+ boutons on 10 M2+ cells in 5 sections from 3 VGAT^Cre^ mice (2% PFA); (10) GFP+ and PV+ somata in 3 sections from 3 PV^Cre^ mice (2% PFA); (11) GFP+ and PV+ boutons in 3 sections from 2 PV^Cre^ mice (2% PFA); (12) GFP+ and VGAT+ boutons on 10 M2+ cells in 4 sections from 2 PV^Cre^ mice (2% PFA); (13) GFP+ boutons on 23 M2+ cells in 7 sections from 2 PV^Cre^ mice (2% PFA); (14) GFP+ and mGluR8a+ boutons on 5 M2+ cells in 3 sections from 2 PV^Cre^ mice (2% PFA).

#### Horseradish peroxidase (HRP) reactions

For high-contrast neurobiotin visualisation, we performed avidin–biotin combined horseradish peroxidase (HRP) reactions with 3,3′-diaminobenzidine (DAB) as chromogen and either 0.002% wt/vol hydrogen peroxide (H_2_O_2_) as substrate, or H_2_O_2_ generated continuously from glucose by glucose oxidase (Sigma-Aldrich). The detailed methods have been published previously (Lasztoczi et al. 2011). After the DAB reactions, sections were treated with osmium tetroxide (0.1–1%) in 0.1 M PB, followed by graded dehydration in ethanol (70–100%) and propylene oxide. For EM, sections were contrast enhanced by uranyl acetate (1% wt/vol, TAAB, for 35 min) added to 70% ethanol. At the end, all sections were embedded in epoxy resin (Durcupan ACM Fluka, Sigma-Aldrich) and polymerised (2 day at 60 °C).

#### Visualisation of EGFP+ cells and PV immunolabelling for combined light- and electron microscopy

After blocking of nonspecific antibody binding sites, two methods of indirect primary antibody detection in combination with DAB histochemistry were used to visualise EGFP+ cells. Most sections (including RT25 s59) were incubated in TBS-Tx containing anti-EGFP primary antibody raised in rabbit (2 nights at 4 °C) recognised by biotinylated anti-rabbit secondary antibody raised in donkey (overnight at room temperature) followed by ABC complex in TBS (2 days at 4 °C) and visualised using DAB (0.05%) in combination with nickel–ammonium sulphate (0.5%) in the presence of H_2_O_2_ (0.001%, for 20 min). Another two sections (RT25 s57, s58) selected for electron microscopy (EM) were processed with freeze–thaw permeabilisation and then were incubated in TBS without detergent containing anti-EGFP and anti-PV primary antibodies (for 40 h at 4 °C) raised in different species. First, biotinylated anti-rabbit secondary antibody and ABC complex were used to detect EGFP in combination with DAB and nickel–ammonium sulphate (black) using the glucose oxidase method for H_2_O_2_ generation. Visualisation of PV continued with a HRP-conjugated anti-mouse antibody in combination with DAB only (brown) in the presence of H_2_O_2_. After the DAB reactions, all sections were treated with osmium tetroxide (1% for EM or 0.3% the rest, for 1 h) in 0.1 M PB, followed by graded dehydration and mounting (see above).

#### Microscopy

The immunohistochemical reactions were first evaluated using wide-field epifluorescence microscopy using either a Leitz DMRB microscope (Leica, Germany) with epifluorescence illumination (Hg arc lamp, HBO, Osram), optimally selected dichroic mirrors and PL Fluotar 20×/0.5 and 40×/0.7 objectives or an AxioImager.Z1 microscope (Carl Zeiss, Germany) with LED illumination and DIC M27 Plan-Apochromat 40×/1.3 oil, 63×/1.4 oil objectives. For documentation and qualitative evaluation of the reactions, images were taken using either a Hamamatsu Photonics ORCA ER CCD camera (C4747-95; Japan) and Openlab v.5.5.0 software (Improvision, UK) or AxioCam HRm CCD camera and AxioVision 2009, Rel.4.8.1 software (Carl Zeiss, Germany), respectively.

Confocal microscopy was performed using the AxioImager.Z1 microscope (Carl Zeiss) equipped with LSM 710 scan head and an additional alpha 20×, 40×, 63 Plan-Apochromat 100×/1.46 oil objective. Image acquisition and analysis was done using ZEN 2008 software, v5.0 (Carl Zeiss). Tracks were switched frame by frame in sequence of increasing excitation wavelengths. Output signals from four subsequent line scans were averaged. Pinhole sizes were adjusted to near 1 Airy Unit (AU) and were slightly varied to produce the same optical slice thickness (typically 0.7 µm) in all channels. Pixel size and numbers were adjusted according to the Nyquist sampling theory. Channel separation was tested by systematic cross-excitation and detection between the channels.

Conventional transmitted light microscopy was performed using either a Leitz Dialux22 microscope (Leica) equipped with NPL Fluotar 10×/0.3, 25×/0.55, 40×/0.78 and PL Apo 100×/1.32 oil objectives or the AxioImager.Z1 microscope (Carl Zeiss) from above. In the case of the former, images were taken using Canon EOS 40D camera controlled by an EOS Canon Utility software v2.9.0.0.

For all imaging modalities, exposure times were individually set for each image depending on the signal strength and the recorded wavelength. Further editing was only done to brightness and contrast levels and was applied for the entire frame in the whole stack and for each channel separately. To reduce noise and enhance contrast levels images were filtered (median algorithm, kernel size *X*, *Y* = 3; kernel size *Z*, channel = 1). When needed, stacks were maximum intensity projected along the *Z*-axis.

#### Serial section electron microscopic quantification of postsynaptic target profiles

Sections selected from a recorded and neurobiotin-labelled cell (D37r) and an EGFP+ cell (RT25) were processed for serial section electron microscopy (see above). Representative areas of axonal field in the CA1 area were re-embedded and ultrathin serial sections (~ 60 nm) were cut and mounted on single-slot, Pioloform-coated copper grids for conventional transmission electron microscopy (Philips CM100, Gatan UltraScan 1000 CCD camera). We identified synapses as Gray’s type I (often called asymmetrical) and type II (often called symmetrical) based on their fine structure; type I synapses having a thick postsynaptic density, whereas type II synapses are characterised by a thin postsynaptic density (Gray 1959). All labelled boutons cut at the section plane were followed in serial sections to locate synaptic junctions between them and their postsynaptic targets in strata oriens, pyramidale and radiatum. Electron microscopic identification and classification of targeted profiles in the hippocampus was according to established criteria (Gulyás et al. 1999; Megias et al. 2001) and by following postsynaptic dendrites in consecutive sections. Briefly, in stratum radiatum and oriens, pyramidal cells of the CA1 region have spiny dendrites and receive type I synapses exclusively on their dendritic spines, whereas their dendritic shafts are innervated mostly by type II synapses. In contrast, the overwhelming majority of hippocampal interneurons have aspiny or sparsely spiny dendrites, which receive type II and also type I synapses onto their shafts (Takács et al. 2012). In addition, pyramidal cell dendrites have frequent electron-opaque clumps of dense material (50–100 nm) associated with the endoplasmic reticulum. Such dense bodies have not been observed in interneurons. These features were extracted when postsynaptic dendrites were followed in sufficient number of serial sections. If too few sections were available, or the postsynaptic dendrite was a small profile that did not receive any synapse, the parent neuron remained unidentified. Note that this classification criterion may lead to an underestimation of interneuron dendrites and a corresponding overestimation of pyramidal cell dendrites. First, interneuron dendrites may get misclassified as originating from pyramidal cells because of their spines (Takács et al. 2012). Moreover, the density of type I synapses on interneuron dendrites may be low, in which case the short series studied may not reveal such a synapse rendering the shaft unidentified.

#### Single cell reconstruction and quantitative morphological analyses

The soma, dendritic tree and representative parts of the axon of an EGFP+ cell (RT25) were manually traced from HRP-reacted (see above) sections (*n* = 3, s57–s59) using a microscope with drawing tube (Leitz Dialux22, Leica) and PL Apo 100×/1.32 oil immersion objective.

The neurobiotin-labelled trilaminar cell (D37r) was digitally reconstructed in 3D and analysed using Neurolucida (MBF Bioscience), on a Nikon Eclipse 80i transmitted light microscope and Lucivid microdisplay (MBF Bioscience) in continuous mode equipped with a VC Plan Apo 100×/1.4 oil immersion objective. Fifty-two coronal sections in consecutive series were reacted with HRP, treated with osmium tetroxide and embedded in resin. Because three sections were lost during processing, neuronal processes were traced from 49 sections and, for the gaps, fragments of axon and dendrite were copied and scaled in *X*, *Y* and *Z *to the right lengths to connect with branches from two neighbouring sections. This extrapolated reconstruction was used for laminar quantification of dendritic and axonal lengths. Individual axonal fragments were split at their crossing points of the laminar boundaries and colour coded accordingly. The pre-spliced lengths were multiplied by the factor 52/49 to estimate the lengths that would have been present in the full cell, had the three sections not been lost.

Tissue shrinkage due to histological processing was corrected (factors reported as mean ± standard deviation) by expanding all elements within individually traced sections in *X*-, *Y*-, and *Z*-dimensions according to criteria we have previously published (Tukker et al. 2013). Briefly, sample sections (*n* = 31) incubated in 4′,6-diamidino-2-phenylindole (DAPI) were measured for thickness before (i.e. wet) and after (i.e. embedded) either treatment with TBS-TX (*n* = 3 sections) or F-T (*n* = 28 sections) using a 63×/1.4 objective and epifluorescence illumination. The thickness (*Z*) was calculated from the digital readout of a closed-loop *z-*stage of a motorised microscope (AxioImager.Z1, Carl Zeiss). Shrinkage correction of embedded sections was started by expanding elements within TBS-TX sections in *X* and *Y* to the size of those in F-T sections (1.1 ± 0.04 correction factor) enabling the alignment and matching of the processes. Next, the thickness of each embedded section was restored to that before treatment using correction factors (1.4 ± 0.3 for TBS-TX; 1.1 ± 0.1 for F-T) obtained by dividing measured wet thicknesses by those embedded. For TBS-TX sections that had no wet thickness measurements (*n* = 9), the correction factor (1.4 ± 0.2) was estimated using the mean wet section thickness from all the measured sections (73.0 μm). For F-T sections that had no wet measurements (*n* = 14), the average factor from the measured F-T sections was used. Finally, all sections were aligned and individually scaled up to wet size in *X* and *Y* by applying the published correction factor (1.04) calculated from measurements of sections with the same type of processing (Tukker et al. 2013).

## Results

### GABAergic trilaminar cells in CA1 and CA3 of rat and mouse hippocampus

**Fig. 1:**
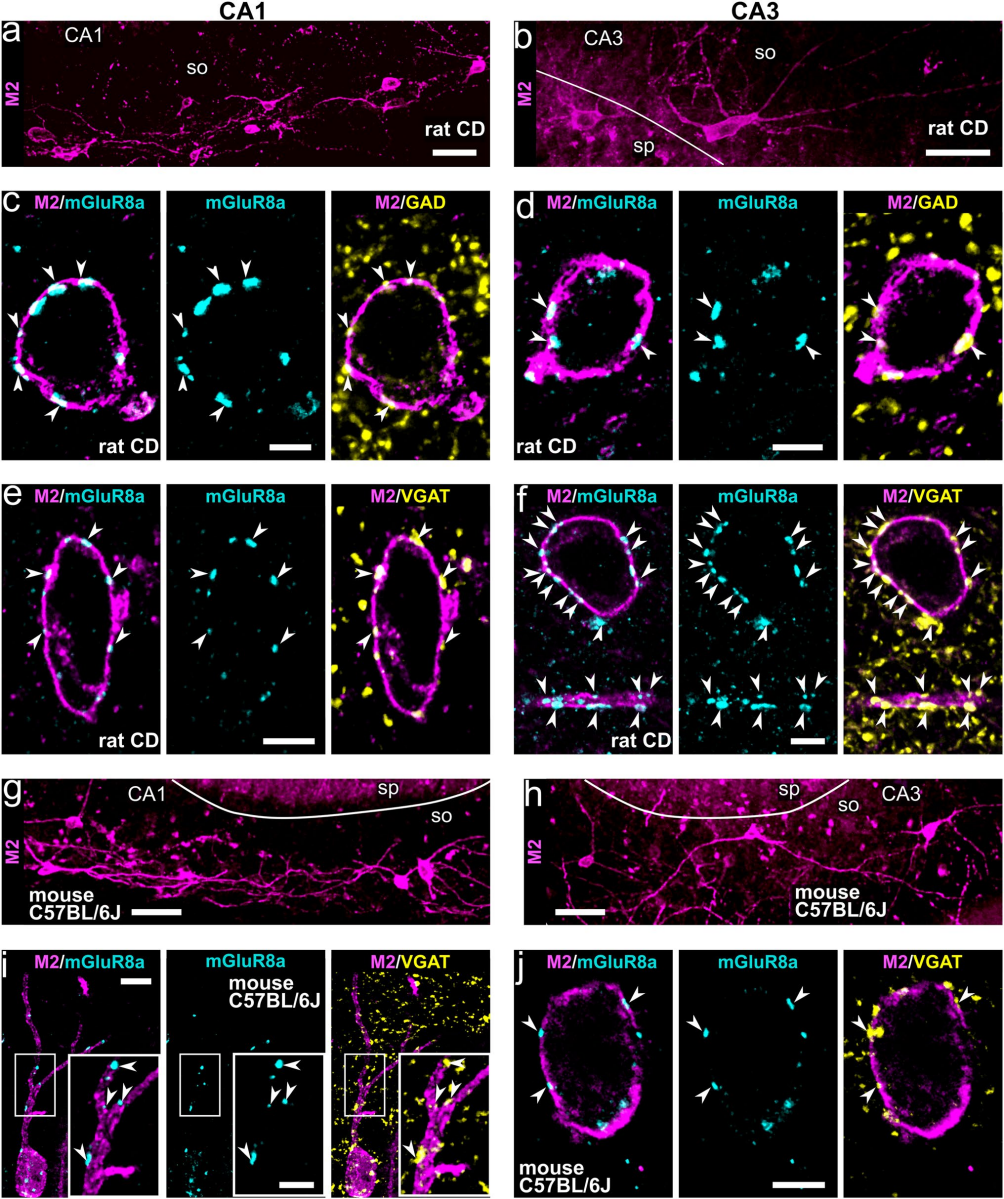
Neurons immunopositive for M2 receive inputs from mGluR8a+ presynaptic terminals, which are mostly GABAergic in areas CA1 and CA3 in rat (**a**–**f**) and mouse (**g**–**j**). **a**,** b** In stratum oriens of the rat CA1 and CA3 (maximum intensity projections, z stacks, heights 21.3 μm and 13.4 μm, respectively), the somato-dendritic membrane of some non-pyramidal cells is strongly M2+. **c**–**f** Trilaminar cells in the rat CA1 (**c** maximum intensity projection, z stack, height 0.9 μm; **e** confocal microscopic single optical section, 0.4 μm) and CA3 (**d** confocal microscopic single optical section, 0.5 μm; **f** maximum intensity projection, z stack, height 1.1 μm) are innervated by mGluR8a+ terminals co-expressing GAD or VGAT (arrowheads). **g**,** h** Neurons immunopositive for M2 in stratum oriens of the mouse CA1 and CA3 (maximum intensity projections, z stacks, heights 38.9 μm and 27 μm, respectively). **i**,** j** Trilaminar cells in the mouse CA1 and CA3 (maximum intensity projection, z stack, height, 3 μm; and confocal microscopic single optical section, 0.4 μm, respectively) are innervated by mGluR8a+ terminals co-expressing VGAT (arrowheads). CD, Sprague–Dawley; so, stratum oriens; sp, stratum pyramidale; + , immunopositive; scale bars 50 μm in **a**,** b**,** g**,** h** 5 μm in **c**–**f**,** j** and inset of **i **10 μm in **i**

**Fig. 2:**
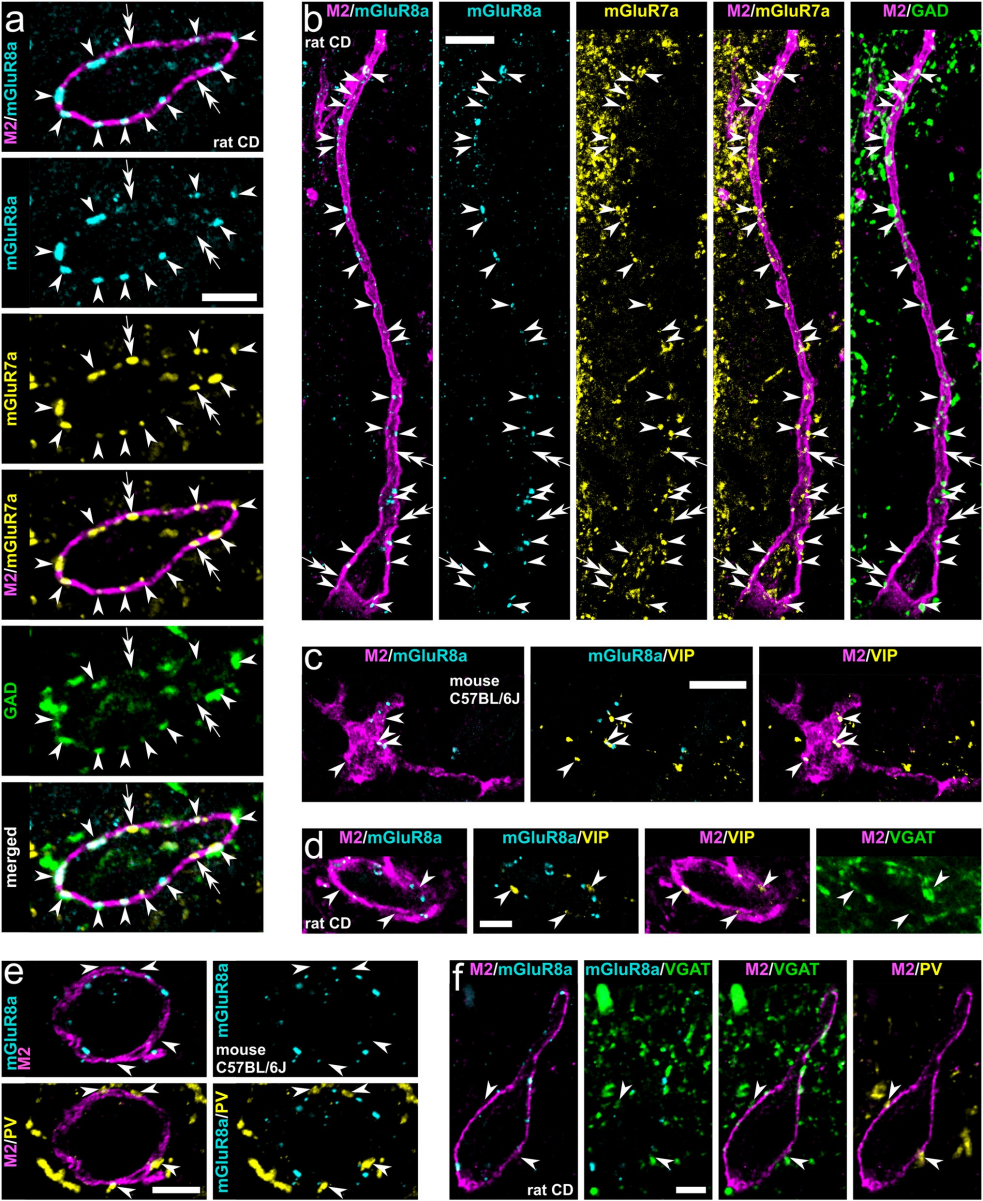
Molecular characterisation of inputs to hippocampal trilaminar neurons in rat (**a**,** b**,** d**,** f**) and mouse (**c**,** e**). **a**,** b** Group III metabotropic glutamate receptors mGluR7a and mGluR8a are co-expressed in the majority of GABAergic GAD+ boutons (arrowheads) targeting trilaminar cells in the CA3 and CA1 (maximum intensity projections, z stacks, heights 0.7 μm and 3.5 μm, respectively). Note also some mGluR7a+/GAD−/mGluR8a− input terminals (double arrows). **c**,** d** Trilaminar cells are innervated by mGluR8a− GABAergic terminals (arrowheads) co-expressing VIP and VGAT (maximum intensity projections, z stacks, heights 1.7 μm). **e**,** f** Trilaminar cells are innervated by mGluR8a− GABAergic terminals (arrowheads) co-expressing PV and VGAT (maximum intensity projection, z stack, height 2.3 μm; and confocal microscopic single optical section, 0.3 μm). CD, Sprague–Dawley; +, immunopositive; −, immunonegative; scale bars 5 μm in **a**, **d**–**f** 10 μm in **b**,** c**

**Fig. 3:**
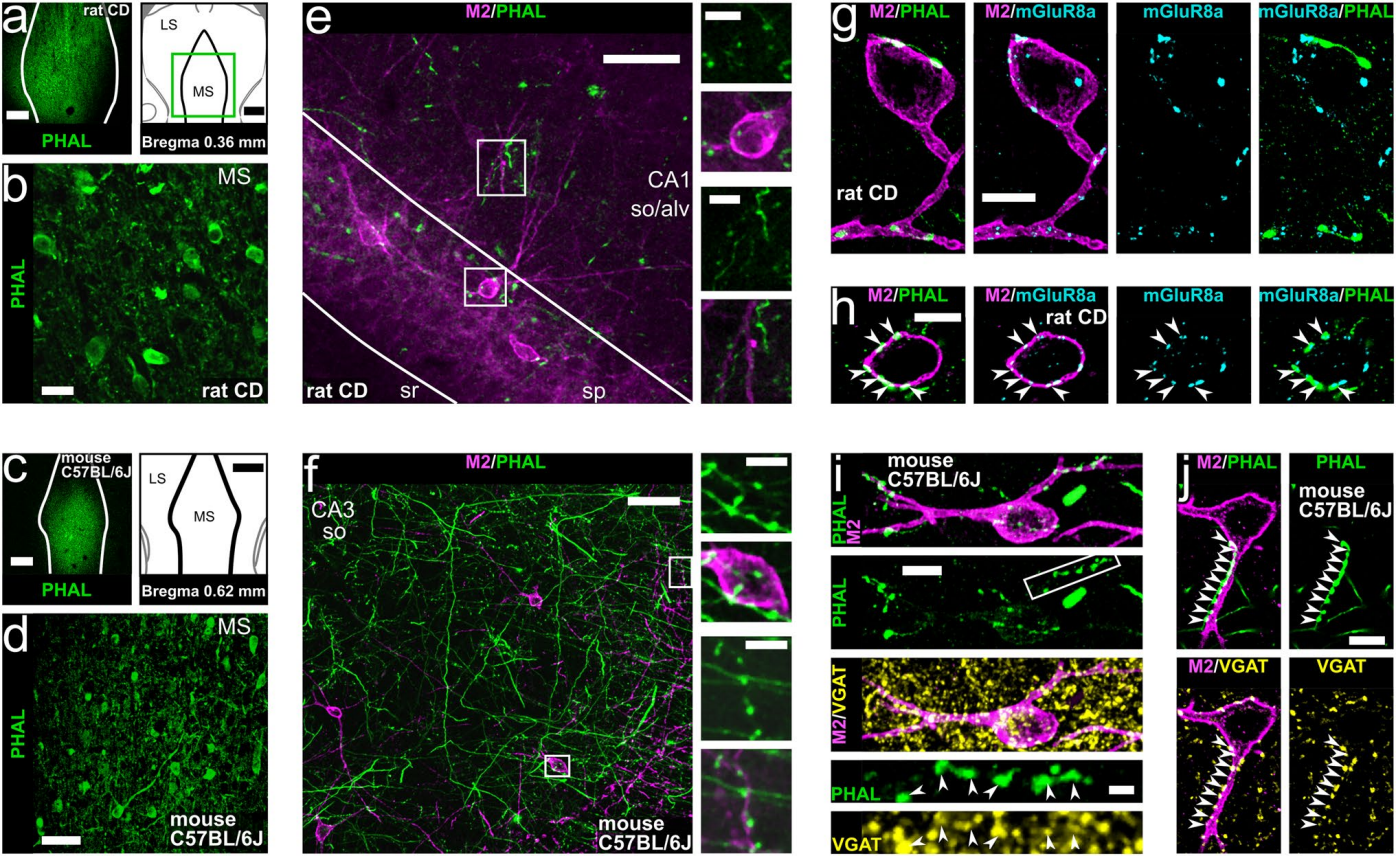
Hippocampal trilaminar cells are innervated by neurons of the medial septum. **a**–**d** Injection sites of the anterograde tracer PHAL (left in **a**–**c**, single optical sections, 20.8 μm) and tracer-labelled neurons in the medial septum of rat and mouse (**b**, **d**, maximum intensity projections, z stacks, heights 5.2 μm and 7.8 μm, respectively). **e**–**f** Axonal projections of PHAL-labelled medial septal neurons in areas CA1 and CA3 (maximum intensity projections, z stacks, heights 12.1 μm and 3.7 μm, respectively) in apposition to cell bodies and dendrites of M2+ neurons (insets). **g**–**h** The M2+ neurons postsynaptic to medial septal axon terminals are densely innervated by mGluR8a+ boutons identifying them as trilaminar cells (maximum intensity projections, z stacks, heights 3 μm and 0.8 μm). Note mGluR8a expression in some of the medial septal input terminals (arrowheads, **h**). **i**–**j** Trilaminar cells postsynaptic to medial septal axon terminals are densely innervated by GABAergic VGAT+ boutons (**i** maximum intensity projection, z stack, height 9.1 μm). Note VGAT expression in some of the medial septal input terminals next to trilaminar cells (**j** arrowheads, confocal microscopic single optical section, 0.5 μm). SD, Sprague–Dawley; MS, medial septum; LS, lateral septum; so, stratum oriens; alv, alveus; sp, stratum pyramidale; sr, stratum radiatum; +, immunopositive; scale bars 500 μm in **a**, 25 μm in **b**, 250 μm in **c**, 50 μm in **d**–**f**, 10 μm in **g**–**j** and insets of **e**,** f**

**Fig. 4:**
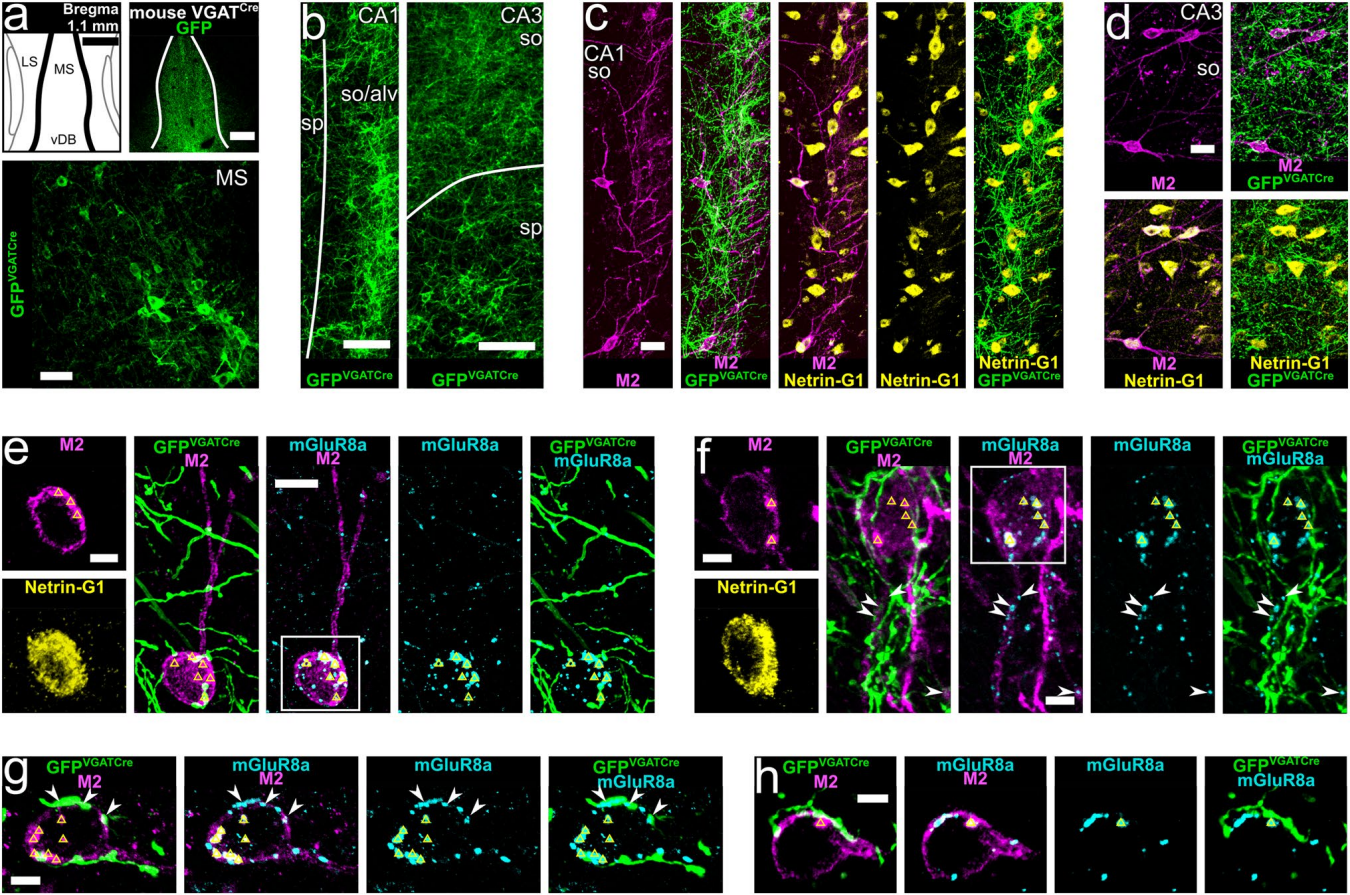
GABAergic neurons of the medial septum innervate hippocampal trilaminar cells. **a** Viral injection site and expression of GFP (top right, single optical section, 20.8 μm) together with labelled GABAergic neurons in the medial septum of a VGAT^Cre^ mouse (GFP^VGATCre^+ ; bottom, maximum intensity projection, z stack, height 13 μm). **b**–**d** GFP^VGATCre^+ medial septal axons in areas CA1 and CA3 in apposition to cell bodies and dendrites of M2+/Netrin-G1+ neurons (maximum intensity projections, z stacks, heights 35.9 μm, 16.2 μm and 27.2 μm). **e–h** M2+/Netrin-G1+ neurons postsynaptic to GFP^VGATCre^+ medial septal boutons innervated by mGluR8a+ terminals identifying them as trilaminar cells (maximum intensity projections, z stacks, heights 10.8 μm, 5.2 μm, 5.4 μm and 4.9 μm). Note mGluR8a expression in some of the GFP^VGATCre^+ medial septal input terminals (arrowheads). In most neuronal somata we detected high levels of lipofuscin (triangles). MS, medial septum; LS, lateral septum; vDB, vertical diagonal band; so, stratum oriens; alv, alveus; sp, stratum pyramidale;+ , immunopositive; scale bars 250 μm in top of **a **50 μm in bottom of **a **100 μm in **b**, 25 μm in **c**,** d**, 10 μm in **e**, 5 μm in **f–h**, and insets of **e**,** f**

**Fig. 5:**
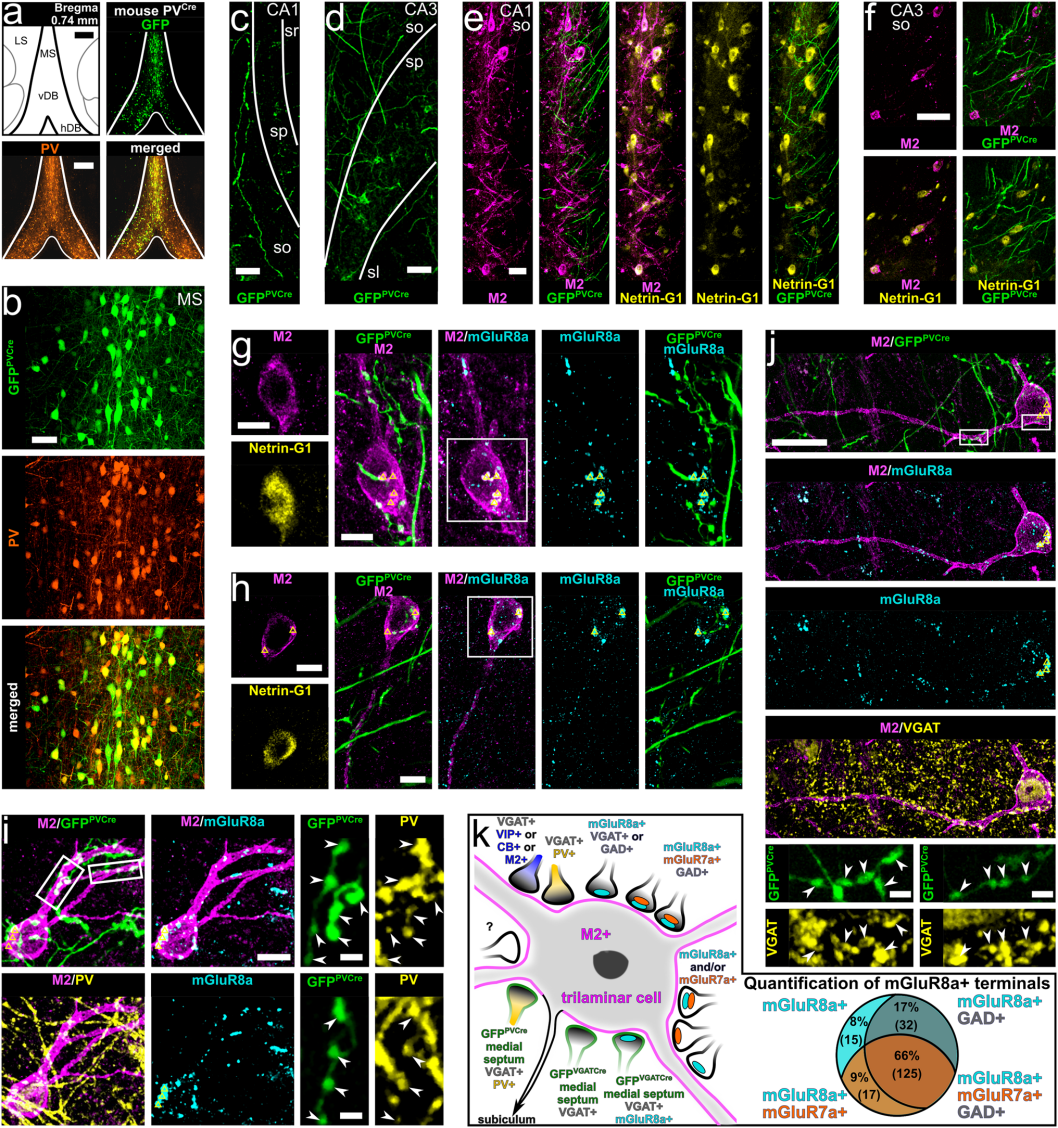
Medial septal neurons expressing PV innervate trilaminar cells in the hippocampus. **a** Viral injection site and expression of GFP together with PV immunoreactivity in the medial septum of a PV^Cre^ mouse (single optical section, 20.8 μm). **b** Co-expression of PV and GFP in neurons of the medial septum (GFP^PVCre^+ ; maximum intensity projection, z stack, height 19.8 μm). **c**–**f** Axonal projections of GFP^PVCre^+ medial septal neurons in areas CA1 and CA3 in apposition to cell bodies and dendrites of M2+/Netrin-G1+ neurons (maximum intensity projections, z stacks, heights 31.2 μm, 31.2 μm, 11.9 μm and 6.8 μm). **g**–**i** M2+/Netrin-G1+ neurons postsynaptic to GFP^PVCre^+ medial septal terminals (arrowheads) innervated by mGluR8a+ boutons identifying them as trilaminar cells (maximum intensity projections, z stacks, heights 4.6 μm, 4.2 μm and 9.2 μm). Expression of PV confirmed in the GFP^PVCre^+ medial septal terminals (**i**). In most neuronal somata we detected high levels of lipofuscin (triangles). **j** Expression of VGAT in the GFP^PVCre^+ medial septal terminals (arrowheads) targeting hippocampal trilaminar cells (maximum intensity projection, z stack, height 5.3 μm). **k** Summary schematic diagram of the identified synaptic inputs innervating M2+ trilaminar cells. Right: quantification of mGluR7a and GAD in mGluR8a+ terminals targeting trilaminar cells. MS, medial septum; LS, lateral septum; vDB, vertical diagonal band; hDB, horizontal diagonal band; so, stratum oriens; sp, stratum pyramidale; sl, stratum lucidum; sr, stratum radiatum; + , immunopositive; scale bars, 250 μm in **a**, 50 μm in **b**–**d**,** f**, 25 μm in **e**,** j**, 10 μm in **g**–**i** and insets of **g**,** h**, 2.5 μm in insets of **i**,** j**

Non-pyramidal neurons with high levels of M2 expression in their somato-dendritic membrane can be visualised in all areas of the rat and mouse hippocampus (Fig. 1a, b, g, h; Hájos et al. 1997; Jinno et al. 2007). Trilaminar cells form one subpopulation of these neurons identified in stratum oriens/alveus in the CA1 area in rat with very dense mGluR8a+ input synapses and long-range projecting axons innervating the subiculum (Ferraguti et al. 2005; Sik et al. 1995). By performing high-resolution quantitative immunohistochemical analyses of M2/mGluR8a-labelled neuronal connections (Figs. 1, 2, 3, 4, 5), we have established the presence of molecularly identified trilaminar cells also in the CA3 area in rat (Figs. 1b, d, f, 2a) and we investigated their distribution in mouse.

Neurons with molecular features of trilaminar cells were consistently found in stratum oriens/alveus in the mouse CA1 and CA3 areas (Figs. 1g–j, 2c, e) and their proportion was similar (*n* = 84 cells; *χ*^2^(1) = 0.01, *p* = 0.921) to that in the rat (16%; Figs. 1a–f, 2a, b, d, f), namely six out of thirty-five (17%; *n* = 3 C57BL/6J mice; in CA1 and CA3) strongly M2+ cells were heavily decorated by at least fifteen mGluR8a+ presynaptic terminals along their somata and proximal dendrites. The other M2+ neurons had a lower number and density of mGluR8a+ terminals, or lacked such innervation.

In the mouse, we observed a subpopulation of large M2+ neurons located in stratum pyramidale, radiatum and at the border of radiatum and lacunosum-moleculare, which are not present in the rat hippocampus. In contrast to oriens/alveus neurons, these cells lacked the strong mGluR8a+ inputs. The frequency of occurrence of M2+ cell bodies was higher in the distal CA1 close to the border with the dorsal subiculum and in the proximal CA1 closer to the CA2/3 border, with very high densities across all layers of the CA3 area.

### Molecular characterisation of synaptic inputs to trilaminar cells in rat and mouse

#### The mGluR8a+ presynaptic terminals innervating trilaminar cells are mostly GABAergic

In stratum oriens of both rat and mouse, mGluR8a+ GABAergic boutons are present on somata and proximal dendrites of strongly M2+ neurons in both areas CA1 and CA3 (Figs. 1c–f, i, j, 2a, b). Quantification in the rat revealed that, on average, 89.1% of mGluR8a+ inputs on presumed trilaminar cells are GABAergic and this proportion is similar between neurons in areas CA1 and CA3 (*n* = 841; *χ*^2^(1) = 0.535, *p* = 0.464; Table 1). In the CA1 area, from a total of 514 mGluR8a+ boutons on presumed trilaminar cells, 89.7% were GABAergic expressing either GAD or VGAT (*χ*^2^(1) = 0.570, *p* = 0.45; Table 1). In the CA3 area, from a total of 327 mGluR8a+ boutons, we identified 88.1% to be GABAergic expressing either GAD or VGAT (*χ*^2^(1) = 3.518, *p* = 0.061; Table 1).

**Table 1:** The mGluR8a+ presynaptic terminals innervating trilaminar cells are mostly GABAergic

Area	Rat code (number of cells)	Number of mGluR8a+ boutons			%
		Total (per cell)	GAD+	VGAT+	
CA1	KD250608 (10)	256 (8–55)	227	NA	88.7
	KD201009K (3)	72 (7–36)	NA	59	81.9
	KD201009L (3)	79 (15–32)	NA	75	94.9
	KD201009M (3)	107 (20–51)	NA	100	93.5
	Total	514	227	234	89.7
CA3	KD250608 (3)	71 (13–33)	58	NA	81.7
	KD201009K (3)	89 (20–40)	NA	79	88.8
	KD201009L (3)	167 (48–66)	NA	151	90.4
	Total	327	58	230	88.1

+ , immunopositive; NA, not applicable

#### Co-expression of mGluR7a and GAD in mGluR8a+ terminals presynaptic to trilaminar cells

Next, we probed the co-existence of mGluR7a, mGluR8a and GAD in the boutons innervating somata and proximal dendrites of trilaminar cells in the rat (Figs. 2a, b, 5k; Table 2). From a total of 189 mGluR8a+ boutons, 66.1% were GABAergic and mGluR7a+ (Table 2). Of all the GABAergic mGluR8a+ terminals (*n* = 157, 83.1%), the majority (79.6%) were mGluR7a+ (*n* = 125; Table 2). Some boutons (8.9%) co-expressed mGluR8a and mGluR7a but were GAD−. Only very few mGluR8a+ terminals (7.9%) were both GAD− and mGluR7a−. Moreover, we encountered rare terminals, which lacked mGluR8a and GAD expression but were mGluR7a+ (Fig. 2a, double arrows).

**Table 2:** Quantification of mGluR8a, mGluR7a and GAD in terminals innervating trilaminar cells

Rat code (number of cells)	Number of mGluR8a+ boutons					
	Total (per cell)	GAD+	GAD+ mGluR7a+	GAD+ mGluR7a−	GAD− mGluR7a+	GAD− mGluR7a−
KD201009K (3)	54 (14–25)	45	31	14	4	5
KD201009L (3)	92 (22–36)	77	71	6	9	6
KD201009M (3)	43 (9–21)	35	23	12	4	4
Total (%)	189	157 (83.1)	125 (66.1)	32 (16.9)	17 (8.9)	15 (7.9)

+ , immunopositive; −, immunonegative

#### Other GABAergic terminals presynaptic to trilaminar cells

**Fig. 6:**
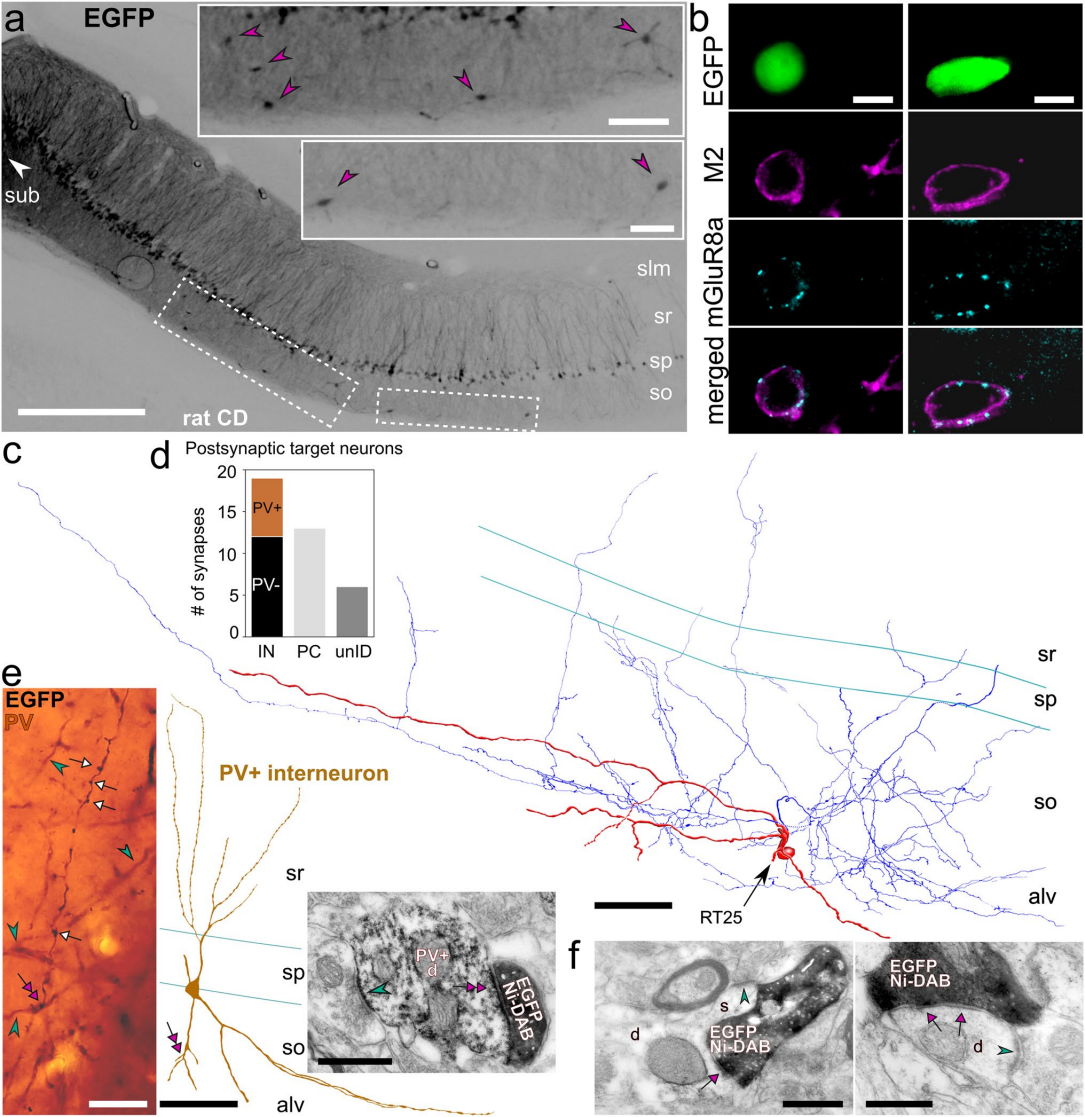
Retrogradely labelled hippocampo-subicular projecting trilaminar neurons in stratum oriens of the rat CA1 area. **a** Neurons in the CA1 area expressing EGFP following the injection of a recombinant adenovirus vector into subiculum (left, white arrowhead). Note some putative GABAergic neurons (insets, magenta arrowheads) retrogradely labelled together with the majority of CA1 pyramidal cells (contrast inverted epifluorescence images). **b** Hippocampo-subicular projecting EGFP-labelled cells in stratum oriens co-expressing M2 and decorated by mGluR8a+ terminals (epifluorescence micrograph). **c** Single-section reconstruction of soma with dendrites (red, section thickness 70 μm) and local axon collaterals (blue) of one EGFP-labelled GABAergic neuron co-expressing M2 and innervated by mGluR8a+ terminals (**b** left). **d** Postsynaptic target distribution of the EGFP-labelled cell reconstructed in **c**. **e***left* Axon collateral with boutons (white arrows) of the EGFP-labelled cell (black, HRP with nickel-DAB) reconstructed in **c** and PV+ dendrites (cyan arrowheads, brown, HRP with nickel-free DAB) in stratum oriens (transmitted light micrograph). Note the contact (magenta double arrow) between one EGFP-labelled bouton and one dendrite of a local PV+ interneuron (middle, reconstruction of soma and dendrites). Right: electron micrograph of the same EGFP-labelled bouton (black, HRP with nickel-DAB) making a type II synapse (magenta double arrow) with the PV+ interneuron dendrite (HRP with nickel-free DAB) also receiving a type I synapse (cyan arrowhead) of unknown origin. **f** Electron micrographs of EGFP-labelled boutons (black, visualised using HRP reaction and nickel-DAB as chromogen) of the cell reconstructed in **c** making type II synapses (magenta arrows) with a pyramidal cell dendrite (left, **d**) and an interneuron dendrite (right, **d**). Note the type I synapses (cyan arrowheads) received onto the spine (left, s) emanating from the dendrite and onto the shaft (right), respectively, identifying the postsynaptic targets as pyramidal cell and interneuron. CD, Sprague–Dawley; IN, interneuron; PC, pyramidal cell; unID, unidentified; + , immunopositive; −, immunonegative; slm, stratum lacunosum-moleculare; sr, stratum radiatum; sp, stratum pyramidale; so, stratum oriens; alv, alveus; sub, subiculum; scale bars 0.5 mm in **a**, 100 μm in insets of **a** and for reconstructions in **c**,** e**, 10 μm in **b** and for epifluorescence images in **c**, 0.5 μm in **d** and for electron micrograph in **e**, 25 μm for light micrograph in **e**

In the rat, some VIP+ terminals in apposition with trilaminar cells are GABAergic VGAT+ (Fig. 2d). Also, we verified the co-existence of VIP and mGluR8a+, although in only 5% of mGluR8a+boutons (Fig. 6c; see section below and Ferraguti et al. 2005); the majority of the VIP+ terminals on M2+ cells lacked mGluR8a immunoreactivity. Moreover, we found presynaptic boutons on M2+ cells co-expressing PV and VGAT which lacked immunoreactivity for mGluR8a (Fig. 2f).

In the mouse, in addition to terminals expressing VIP and PV (Figs. 2c, e, 5j), we have also observed calbindin-expressing (CB+) and M2+ terminals in apposition to mGluR8a-decorated M2+ cells in stratum oriens; these boutons also lacked immunoreactivity for mGluR8a (data not shown). When tested, terminals strongly expressing the cannabinoid receptor type 1 (CB1) and most likely originating from cholecystokinin-expressing interneurons were also immunonegative for mGluR8a, irrespective of whether they were next to M2+ dendrites or not (data not shown).

#### Non-GABAergic presynaptic mGluR8a+ terminals

Extensive examination of the approximately 10% mGluR8a+ terminals that appeared to lack GABAergic molecular markers and were in apposition to M2+ somata and dendrites in stratum oriens has shown minimal co-localisation between mGluR8a and vesicular glutamate or acetylcholine transporters (see below). We have not tested for the presence of monoaminergic molecular markers in these boutons.

Although, in rat, we did not find evidence of vesicular glutamate transporter 1 (VGluT1) in mGluR8a+ terminals next to trilaminar cells (*n* = 258 boutons on 3 cells from 3 rats; 72–107 boutons per rat), we did observe the presence of vesicular glutamate transporter 2 (VGluT2) in some mGluR8a+ boutons on M2+ interneuron dendrites in the mouse hippocampus. It remains to be determined which type of M2+ non-pyramidal cell receive the mGluR8a+/VGluT2+ terminals.

Using immunolocalisation of vesicular acetylcholine transporter (VAchT) in the rat, we have confirmed that some cholinergic terminals in stratum oriens co-express mGluR8a and innervate M2+ cells (Ferraguti and Shigemoto 2006). Furthermore, although very few cholinergic mGluR8a+ terminals are in apposition to each individual M2+ neuron, these were all VGAT+ (*n* = 12 boutons on 4 cells in 2 rats; see also (Takács et al. 2018)).

### Hippocampal trilaminar cells receive long-range subcortical inputs from GABAergic neurons of the medial septum

The medial septum contains GABAergic, cholinergic, and glutamatergic neurons which send long-range projections to the hippocampus (Colom et al. 2005; Freund 1989; Hajszan et al. 2004; Joshi et al. 2017; Kimura et al. 1980; Köhler et al. 1984; Salib et al. 2019; Unal et al. 2018, 2015; Viney et al. 2018). Using three different methods of anterograde labelling in rats and mice, together with high-resolution quantitative immunohistochemistry, we show that the majority of GABAergic inputs to trilaminar cells in the hippocampus originate from GABAergic cells in the medial septum as detailed below (Figs. 3, 4, 5; Table 3).

**Table 3:** Medial septal GABAergic terminals innervating trilaminar cells

Mouse line	Animal code (number of cells)	Number of boutons on M2+ neurons		
		GFP^VGATCre^+ (per cell)	mGluR8a+ (per cell)	GFP^VGATCre^+ mGluR8a+ (per cell)
VGAT^Cre^	KH213 (4)	52 (11–18)	83 (17–27)	19 (3–7)
	KH221 (2)	32 (13,19)	57 (24,33)	7 (3,4)
	KH222 (4)	37 (5–12)	97 (18–35)	9 (1–4)
	Total	121	237	35
	Median	12	22	4
	IQR	2	8	2

Mouse line	Animal code (number of cells)	Number of GFP^PVCre^+ boutons on M2+ neurons (per cell)	
PV^Cre^	LK50 (10)	170 (9–27)	
	LK70 (13)	186 (6–28)	
	Total	356	
	Median	15	
	IQR	11	

**+ **, immunopositive; IQR, interquartile range;

#### Medial septal mGluR8a+ terminals innervate trilaminar cells in rat

In rats, we labelled neurons using *Phaseolus vulgaris*-leucoagglutinin (PHAL+) in the medial septum (Fig. 3a, b) and their axon collaterals entering and branching in all layers of the hippocampus (Fig. 3e, g, h). In both the CA1 and CA3 areas, PHAL+ terminals targeted somata and dendrites of M2+ hippocampal cells (Fig. 3e, g, h). Each postsynaptic cell was innervated by numerous PHAL+ boutons, and the M2+ cells included those densely decorated by mGluR8a+ terminals (Fig. 3g, h; *n* = 13 ± 4 terminals, see “Materials and methods”). Some of the PHAL+ septal boutons targeting M2+ cells expressed mGluR8a (Fig. 3h, *n* = 10 out of 13 terminals; see “Materials and methods”) although many were immunonegative for the receptor. These results reveal the existence of an mGluR8a+ medial septo-hippocampal neuron population, some of which innervate trilaminar cells.

#### Medial septal GABAergic terminals innervate trilaminar cells in mouse

To gain neurotransmitter type specificity, we pursued experiments in mice. First, repeating PHAL labelling of medial septal neurons (Fig. 3c, d) and their axon collaterals in hippocampus (Fig. 3f, i, j), we found that their terminals are VGAT+ and innervate M2+ cells (Fig. 3i, j; 19 out of 23 tested on 3 cells in 1 mouse).

Next, performing viral injections into the medial septum of VGAT^Cre^ mice (Fig. 4; *n* = 5 animals; see “Materials and methods”), resulted in the expression of GFP in transfected GABAergic cell bodies, dendrites and axons in medial septum (GFP^VGATCre^+; Fig. 4a). As expected, GFP^VGATCre^+ medial septal axons were found in all layers of the hippocampus (Fig. 4b–h; 97.0% VGAT+; *n* = 164 out of 169 terminals; see “Materials and methods”). Focusing on stratum oriens, we confirmed that M2+ neurons co-expressed Netrin-G1 as reported by single-cell and in situ RNA sequencing (Harris et al. 2018; Qian et al. 2019) and demonstrated following transcriptomic profiling of VIP-LRP cells (Luo et al. 2019). Each neuron was innervated, on average, by 12 ± 2 GFP^VGATCre^+ medial septal terminals along their cell bodies and proximal dendrites in a single section, in both the CA1 and the CA3 areas (Fig. 4c–f; Table 3; median ± interquartile range; *n* = 121 terminals). Furthermore, some such cells postsynaptic to GFP^VGATCre^+ medial septal axon terminals were densely innervated by 22 ± 8 mGluR8a+ boutons per cell in a single section (*n* = 10 cells), identifying them as trilaminar cells (Fig. 4e, f; Table 3; median ± interquartile range; *n* = 237 terminals). Co-expression of mGluR8a was found in 28.9% of the GFP^VGATCre^+ medial septal boutons targeting trilaminar cells (Fig. 4f, g; Table 3; *n* = 35 boutons).

#### Medial septal PV+ GABAergic terminals innervate trilaminar cells in mouse

Viral injections into the medial septum of PV^Cre^ mice (Fig. 5; *n* = 4 animals; see “Materials and methods”) enabled the expression of GFP in transfected PV+ GABAergic cell bodies, dendrites and axons (GFP^PVCre^+; Fig. 5a, b) marking one subpopulation of GABAergic long-range projecting medial septal neurons. PV-immunoreactivity was confirmed in 84.1% of 214 GFP^PVCre^+ somata (see “Materials and methods”) with 57.3% transfection rate (*n* = 180 out of 314 PV-immunoreactive somata; see “Materials and methods”).

In the hippocampus, we observed a high density of virally labelled PV+ septal axons in both areas CA1 and CA3 with a bias towards the latter (Fig. 5c–j; confirming Fig. 8a in Salib et al. 2019). The co-expression of virally delivered GFP and PV was verified in the boutons of these medial septal collaterals (Fig. 5i; 90.7% PV+ ; *n* = 185 out of 204 terminals; see “Materials and methods”). In another set of experiments, we confirmed that these GFP^PVCre^+ septal terminals are GABAergic using VGAT immunoreactivity (Fig. 5j; 98.4% VGAT+; *n* = 183 out of 186 terminals; see “Materials and methods”).

On average, 15 ± 11 axon terminals of the GFP^PVCre^+ medial septal neurons targeted M2+ cells along their cell bodies and proximal dendrites in a single section (Fig. 5e–j; Table 3; median ± interquartile range; *n* = 356 terminals). Some, but not all, such postsynaptic target neurons (Fig. 5g–j) were also observed being densely decorated by 24 ± 10 mGluR8a+ terminals per cell, identifying them as trilaminar cells (Table 3; median ± interquartile range; *n* = 137 terminals; see “Materials and methods”). No co-localisation was found in septal terminals between the GFP^PVCre^ (*n* = 100) and mGluR8a+ (*n* = 137; see “Materials and methods”) on these cells.

In summary (Fig. 5k), the very dense mGluR8a+ input of hippocampal trilaminar cells is mostly GABAergic and these terminals often co-express mGluR7a. Much of this GABAergic input originates from medial septal neurons. Specifically, around one-third of the GABAergic medial septal terminals are mGluR8a+; and mGluR8a immunonegative terminals are largely from medial septal PV+ neurons. Presumably, other local hippocampal GABAergic neurons contribute to innervating trilaminar cells with VIP+, PV+, CB+ and M2+ presynaptic terminals, which are predominantly mGluR8a immunonegative.

### PV+ interneurons are frequent targets of trilaminar cells in rat CA1

We used a retrograde tracing strategy in rats combined with serial section electron microscopy and immunohistochemistry to determine the postsynaptic target neurons of a subiculum-projecting trilaminar cell in the rat CA1 area.

Retrograde viral injections into the subiculum (Fig. 6a; see “Materials and methods”) resulted in the expression of EGFP in pyramidal cells and some non-pyramidal neurons including highly EGFP-expressing cells in the subiculum and lower expression levels in the CA1 (Fig. 6a, insets); confirming the topographical projection patterns of the CA1 pyramidal neurons. Only a small proportion of non-pyramidal cells were labelled in strata oriens and radiatum, indicating the lack of virus spread into CA1. The EGFP+ hippocampo-subicular projecting putative GABAergic neurons were located across all layers but mostly in stratum oriens (79%, *n* = 49/62 cells). Of these, we identified 16% (*n* = 8/49 cells) as trilaminar cells based on co-expression of M2 in the somato-dendritic membranes and very dense innervation by mGluR8a+ terminals (Fig. 6b). The tested mGluR8a-innervated M2-expressing projection neurons were immunonegative for SST, CB and calretinin.

Based on extensive labelling of the dendrites and the axon, we selected one such trilaminar cell for light microscopic reconstruction of its soma, dendrites and local axon collaterals (RT25, Fig. 6c). Electron microscopic examination of the boutons along the axon showed that this trilaminar cell innervated the dendritic shaft of both pyramidal cells and interneurons locally in the CA1 area (Fig. 6d–f; Table 4). Fifty-nine percent of a total of 32 identified postsynaptic targets (*n* = 19/32 targets) were interneuron dendrites (Fig. 6d). This ratio is comparable to previously published results (Ferraguti et al. 2005). In addition, we found that many (37%, *n* = 7/19 targets) of the synaptically targeted interneuron dendrites were PV+ (Fig. 6d, e).

**Table 4:** Postsynaptic target distribution and specificity of trilaminar cells in the CA1 area of rats

Cell ID	Interneuron				Pyramidal cell		Unidentified	
	Total	%	PV+	%	Total	%	Total	%
D37r	16	84	n/a	n/a	3	16	2	10
RT25	19	59	7	37	13	41	6	16

+, immunopositive

### Synaptic organisation and behavioural state-dependent activity of an identified trilaminar cell in the rat

**Fig. 7:**
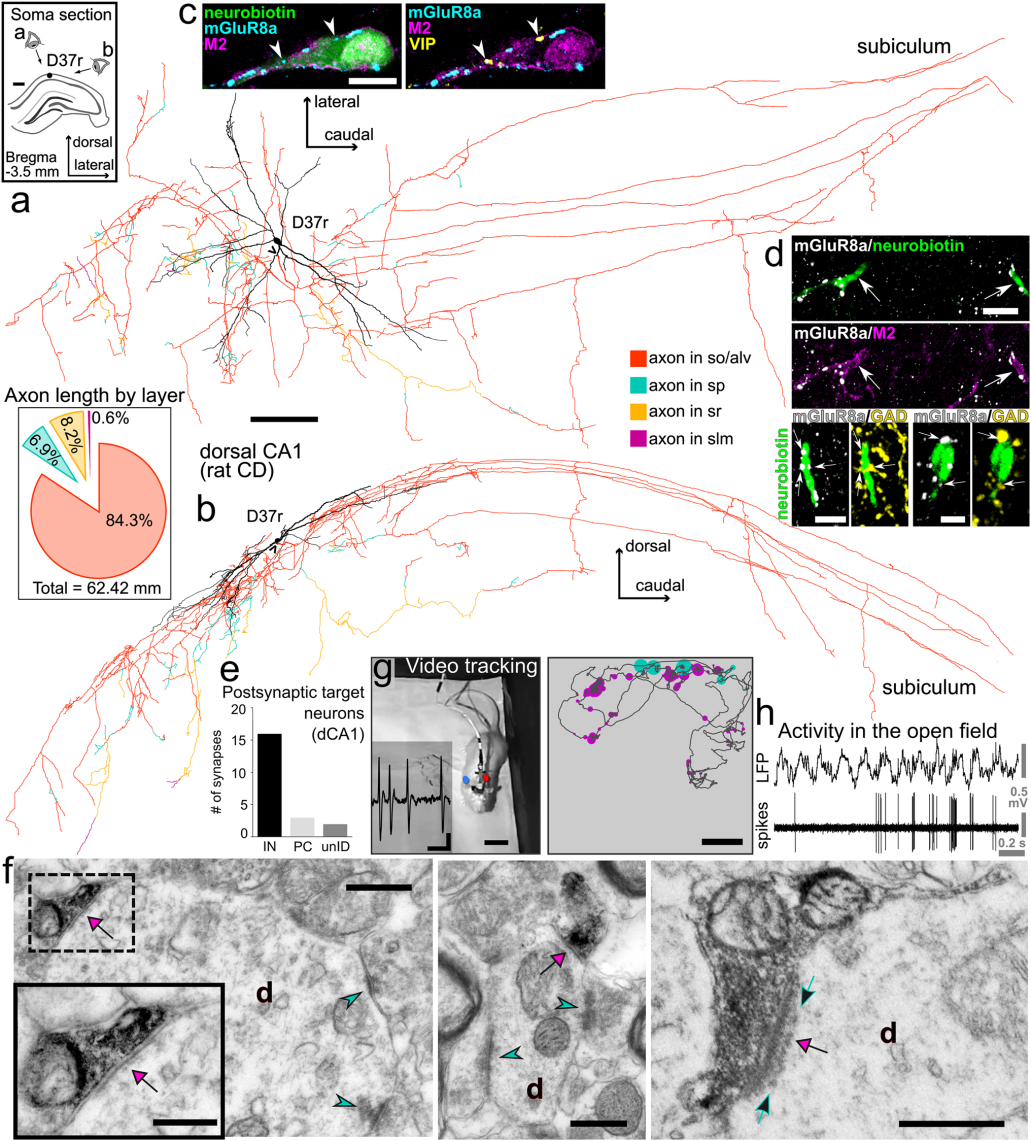
Synaptic organisation and activity during exploration of an identified trilaminar cell (D37r) in the rat hippocampal CA1 area. **a**,** b** Reconstruction of the neurobiotin-filled soma with dendritic tree (black, *n* = 16 70-μm-thick sections) and axon collaterals (colour-coded by layers, *n* = 52 70-μm-thick sections; axon origin, arrowhead) including long-range projections innervating the subiculum; visualisation using transverse (horizontal) plane in **a** and sagittal plane in **b**. Inset in **a** location of the trilaminar cell body (black dot) in stratum oriens of the dorsal CA1 in a coronal section of the right hemisphere. Inset in **b** total axonal length and proportions quantified by layers, showing high preference for stratum oriens/alveus and branching in strata pyramidale and radiatum (colour-coding as in **a**,** b**), but avoiding stratum lacunosum-moleculare. **c** Expression of M2 in the soma and proximal dendrite and dense innervation by mGluR8a+ terminals identify this neuron as a trilaminar cell. Note two mGluR8a+ boutons (arrowheads) also VIP+ (confocal microscopic single optical section, 0.7 μm). **d** Top: two neurobiotin-filled dendrites (arrows) with M2+ plasma membranes receive dense mGluR8a+ input. Bottom: some mGluR8a+ terminals (arrows) in apposition to two trilaminar cell dendrites are GABAergic co-expressing GAD (maximum intensity projections, z stacks, heights 4.3 μm and 7.2 μm). **e** Postsynaptic target distribution of the identified trilaminar cell in the dorsal CA1. **f** Electron micrographs of the neurobiotin-labelled boutons (black, visualised using HRP reaction and nickel-DAB as chromogen) making synapses (magenta arrows) with interneuron dendrites in strata oriens (left, middle, **d**) and pyramidale (right, **d**). Type I synapses (cyan arrowheads) received onto the dendritic shafts identify the postsynaptic targets as interneurons. Note a row of postsynaptic ‘Taxi bodies’ of electron-opaque dots (right, green arrows) co-aligned with the synaptic junction in the interneuron dendrite from the pyramidal layer. **g** Left: video tracking of the rat exploring a familiar open field during extracellular recording of action potentials (inset) and juxtacellular labelling of the trilaminar cell. Right: reconstructed path (dark grey) of the animal with superimposed trilaminar cell activity (dots scaled by firing rate and colour-coded by behavioural state, magenta for movement and cyan for quiet wakefulness) indicate higher firing rate along the top wall. **h** Action potential firing of the identified trilaminar cell during exploration. CD, Sprague–Dawley; so, stratum oriens; alv, alveus; sp, stratum pyramidale; sr, stratum radiatum; slm, stratum lacunosum moleculare; dCA1, dorsal CA1; IN, interneuron; PC, pyramidal cell; unID, unidentified; d, dendrite; +, immunopositive; scale bars, 0.25 mm in **a**, **b**, 0.5 mm in inset of **a**, 10 μm in **c**, **d** top, 5 μm in **d** bottom left, 2.5 μm in **d** bottom right, 0.5 μm in **f**, 0.25 μm in inset of **f**, 0.25 mV vertical, 5 ms horizontal in **g** left, 10 cm in **g** right

**Fig. 8:**
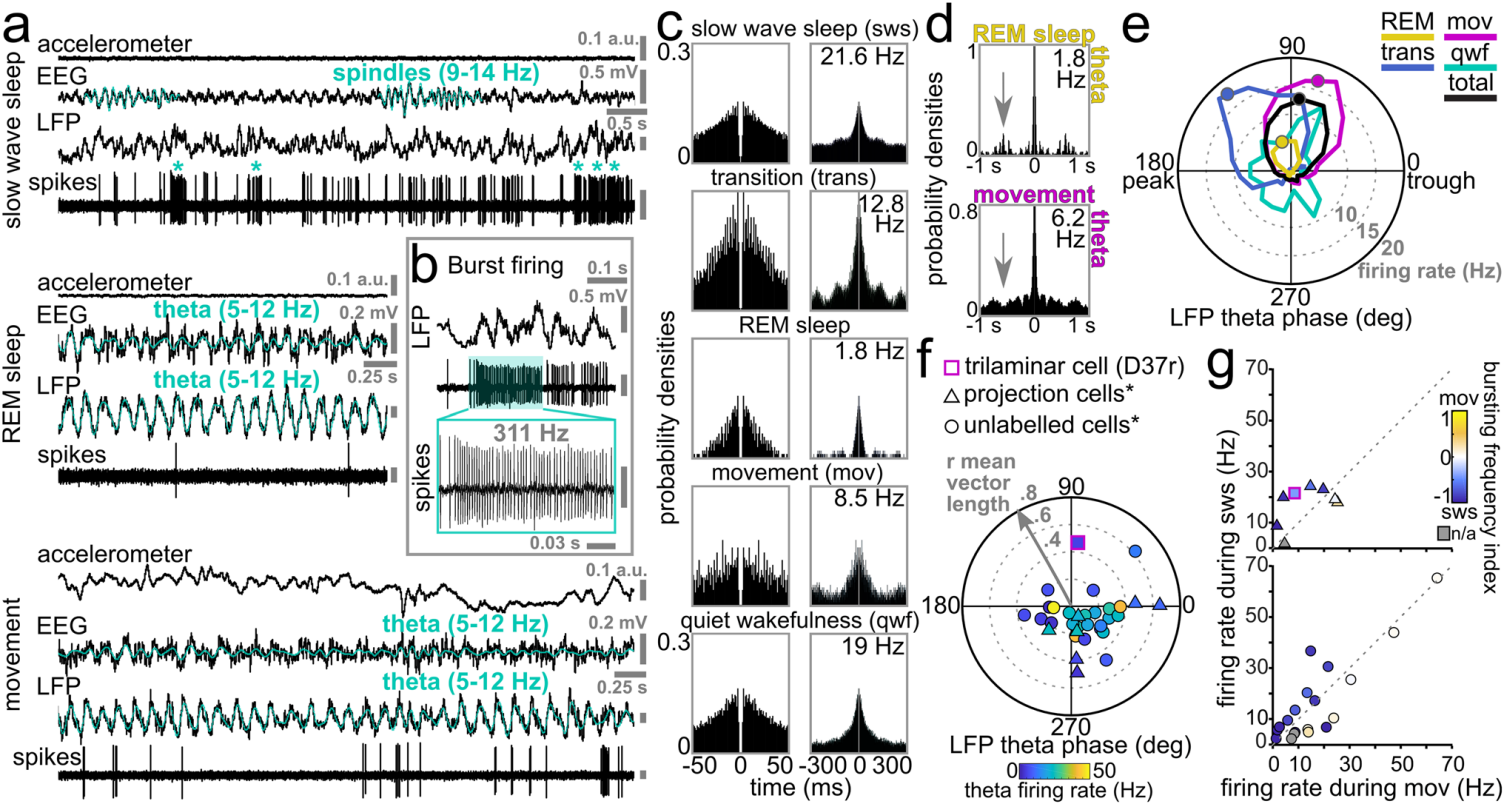
Behavioural state dependent activity of an identified trilaminar cell (D37r) in the rat hippocampal CA1 area. **a** Recording traces of the trilaminar cell activity during slow wave sleep (top) indicated by the occurrence of spindles (cyan) in the cortical EEG. Note irregular spiking with frequent bursts (asterisk) which switched to periodically regular firing with the emergence of theta oscillations (cyan) in both the hippocampal LFP and cortical EEG during REM sleep (middle) and movement (bottom). **b** A burst of action potentials at 311 Hz identifying trilaminar cells in the dorsal CA1. Such bursts lasting up to 200 ms occurred mostly during slow wave sleep and quiet wakefulness, likely associated with similarly high-frequency network events in the LFP e.g. SWRs. **c** Behavioural state-specific action potential autocorrelograms of the trilaminar cell show an early peak which resembles that of pyramidal cells firing complex spike bursts. Mean firing rates are reported in the top right corners. Note the decrease in firing rate of D37r between sleep and movement, the activity becoming minimal during REM. Time axes were limited to 50 ms (left) and 300 ms (right), bin width was set to 1 and 3 ms, respectively. **d** Action potential autocorrelograms during theta oscillations associated with REM sleep and movement demonstrating intermittent trilaminar cell activity skipping many cycles and discharging few action potentials per cycle. Note the peak (arrow) in the top autocorrelogram demonstrating an additional slow (1.7 Hz) modulation of trilaminar cell firing specifically during REM. Time axes were limited to 1 s and bin width was set to 10 ms. **e** Distribution of firing phases (colour-coded by behavioural states) and associated firing rates (dotted circles with grey numbers) of the labelled trilaminar cell during theta oscillations. Mean preferential theta phases (coloured dots) plotted only in episodes of significant coupling to theta cycles. Note strong tuning of the neuron along the ascending slope and the much lower firing rate during REM. **f **Preferred mean firing phase, strength of spike coupling to theta cycles (dotted circles along grey arrow) and mean firing rate during theta epochs (colour-coding of symbols) of the trilaminar cell together with other dCA1 long-range projecting GABAergic neurons (different symbols show neuron categorization). Note the strong coupling of the cell to the ascending slope of theta cycles, 180° out of phase from the majority of projection cells firing along the descending slope. **g** The firing rate of the trilaminar cell as of that of other dCA1 long-range projecting GABAergic neurons was higher during sleep than during movement (same symbols used as in **f**). Colour-coding represents changes in bursting frequency during sleep and movement. *Data from Katona et al. (2017) for comparison

By extracellular single cell recording using glass electrodes in the dorsal CA1 area of freely moving rats (Katona et al. 2017; Lapray et al. 2012), we targeted GABAergic neurons in stratum oriens exhibiting high-frequency (> 200 Hz) bursts of action potentials. At the end of each recording session, we attempted to label the recorded neurons juxtacellularly. Out of 34 recordings in stratum oriens/alveus, we encountered two putative trilaminar cells with the amplitude of their action potentials above the threshold for automatic spike detection and successfully labelled and confirmed one as a subiculum-projecting trilaminar cell (D37r, Figs. 7, 8; Table 5).

**Table 5:** Behavioural state-dependent activity of a juxtacellularly labelled trilaminar cell in rat

	Slow wave sleep	Sleep–wake transition	REM sleep	Movement	Quiet wakefulness	Theta (all cycles)
Mean firing rate (Hz)	21.6	12.8	1.8	8.5	19	5
Median of ISI (ms) (± IQR)	7.4 (17)	3.5 (5.6)	7.7 (447.2)	8.4 (30.6)	6.3 (14.5)	8.9 (114.6)
Autocorrelogram peaks (ms)	3; 113–150; 225–280	3; 135; 270	3; 105; 245	3; 250	3; 150	3; 150; 250
Mean theta phase of firing (°)	NA	130	108	75	NA	84
Mean theta vector length of firing	NA	0.66	0.58	0.50	NA	0.47
Active theta cycles (%)	NA	22	10	27	23	22
Burst probability theta cycles (%)	NA	NA	NA	NA	NA	6.2
Burst frequency (Hz)	2.1	NA	NA	0.8	NA	NA
Burst frequency index [− 1,1]	− 0.43					

IQR, interquartile range; NA, not applicable

#### Input–output synaptic organisation of trilaminar cell D37r

Following neurobiotin labelling of this trilaminar cell in the dorsal CA1 area, the cell was reconstructed (Fig. 7a, b) through 52 consecutive coronal sections of 70 μm thickness each. After correcting for tissue shrinkage, the complete length of dendrites was 8.3 mm, and the recovered axonal arbour was 62 mm in length including long-range projections innervating the subiculum (see “Materials and methods”).

The cell body was located in stratum oriens close to the border with the alveus and the large horizontal dendritic tree was contained within stratum oriens (Fig. 7a, inset). Expression of M2 in the neurobiotin-labelled somatic and dendritic membranes and a dense innervation by mGluR8a+ boutons (Fig. 7c, d) support the identification of this neuron as a trilaminar cell. The mGluR8a+ presynaptic terminals were mostly GABAergic, as shown by co-localization with either GAD (Fig. 7d bottom) or VGAT, and co-expressed low levels of mGluR7a. Furthermore, from a sample of 79 mGluR8a+ boutons in apposition with the soma and proximal dendrite, 5% (*n* = 4) were VIP+ (Fig. 7c); the trilaminar cell soma was VIP immunonegative.

The highest proportion of local collaterals was in stratum oriens/alveus and smaller ramifications in strata pyramidale and radiatum with the exclusion of stratum lacunosum moleculare (Fig. 7b, inset). Using serial section electron microscopic quantification of postsynaptic target profiles, we determined that, similar to RT25 above, this trilaminar cell also made synapses preferentially with interneurons (84%, *n* = 16/19 identified targets; Table 4) and some spiny dendrites, which probably originated from pyramidal cells (Fig. 7e, f). In a small proportion of cases, the identity of the postsynaptic neurons could not be confirmed (Fig. 7e). Often we observed a row of postsynaptic ‘Taxi bodies’ of electron-opaque dots co-aligned with the synaptic junction in the postsynaptic dendrites (Fig. 7f, right; Taxi 1961; Taxi and Babmindra 1972) whose molecular identity remains to be determined. Collaterals of the main axon travelled within stratum oriens or at the border with alveus and the cortical white matter for a distance of 2.8 mm from the soma (40 sections of 70 μm thickness) in the caudo-lateral direction before reaching subiculum where these branched extensively. As subicular terminals could not be visualised due to suboptimal neurobiotin labelling, the identification of postsynaptic target neuron types was not attempted.

#### Trilaminar cell activity patterns in the rat during sleep and wakefulness

During the ~ 9.5-min recording period, the rat was allowed to freely explore and rest in a familiar open field (Fig. 7g, h). The time periods were segmented as movement, quiet wakefulness, slow wave sleep and epochs of rapid eye movement (REM) sleep, facilitating behavioural state-dependent analyses of the trilaminar cell activity (Fig. 8; Table 5).

During *slow wave sleep*, the mean action potential frequency was 21.6 Hz. The irregular low-frequency spiking of the cell was interspersed with frequent bursts at very high frequencies (> 200 Hz) which often co-occurred with events of high network activation in the LFP resembling 130–230 Hz SWRs. (Fig. 8a–c). During *movement*, a very different pattern emerged with periodical regular firing at the much lower mean rate of 8.5 Hz, which decreased further to 1.8 Hz during *REM sleep* (Fig. 8a). The higher firing rate during sleep compared to movement was similar to some identified long-range projecting SST+ cells (Fig. 8c, g; Katona et al. 2017). The minimal activity of the trilaminar cell during REM sleep contrasts with the maximal activation (24.9 Hz) of a recorded long-range projecting SST+ cell (DS17c, Katona et al. 2017). During *quiet wakefulness*, the mean spike rate of the trilaminar cell (19 Hz) was similar to that during slow wave sleep and to the mean spike rates of all projection cells during movement (14.7 ± 17.7 Hz, median ± interquartile range; Fig. 8c, g and Katona et al. 2017).

To evaluate rhythmicity in the trilaminar cell firing, we calculated autocorrelograms (Fig. 8c; Table 5) and discovered behaviour-dependent variability in the spike trains. We extracted the time points of the first and, if present, the second and third peaks within a 300 ms range. We found an early increase in firing at 3 ms during all states, evidence for the high-frequency bursts of the trilaminar cell as reported under urethane anaesthesia (Ferraguti et al. 2005) and resembling the complex spike bursts fired by pyramidal cells. In contrast to the SST+ projection cells (Katona et al. 2017), additional peaks were found between 105 and 150 ms and 225 and 280 ms reporting 7–10 Hz theta- and 4 Hz delta-rhythmic modulation, respectively.

The median inter spike interval (ISI) lengths across behavioural states were consistently less than 9 ms which is specific to this cell type amongst hippocampal interneurons reported so far (Table 5). We detected behavioural state-dependent differences in the ISI distributions with the interquartile ranges extending between 5.6 ms during *sleep–wake transition* and 447.2 ms during *REM sleep*. We monitored the burst firing of the trilaminar cell of three or more action potentials with ISIs shorter than or equal to 12 ms during *movement* and during *slow wave sleep* (Fig. 8a, b). Bursts were common throughout the recording and the bursting frequency was 2.5 times higher during slow wave sleep than during movement as also measured by the negative burst frequency index of − 0.43.

During *movement*, *REM sleep* and periods of *quiet wakefulness*, we detected theta frequency (5–12 Hz) rhythmic network activity in the hippocampal LFP (Fig. 8a; Table 5). During theta oscillations, the trilaminar cell fired with a mean rate of 5 Hz—lower than most SST+ projection cells—and ISI lengths of 8.9 ± 114.6 ms (median ± interquartile range)—much shorter than that of SST+ projection neurons. The cell’s firing was strongly phase-coupled to theta cycles with mean vector length of 0.47 (Fig. 8e, f; Table 5).

We have segmented theta epochs according to different behavioural states and have found differences in the distribution of preferential firing rates and phases (Fig. 8d, e; Table 5). There was a striking 67% drop of the mean firing rate during *REM sleep* compared to other theta periods. Also, the mean preferential theta phases were differently distributed along the ascending slope from 75° during *movement* to more advanced 130° during *sleep–wake transition* periods (Fig. 8e; 0° denotes the trough of the extracellularly recorded LFP theta oscillations). The increased firing of the trilaminar cell during the ascending slope is 180° out of phase as compared to the majority of SST+ long-range projection cells firing along the descending slope (Fig. 8f). The trilaminar cell was not significantly coupled to theta cycles during quiet wakefulness.

Action potential autocorrelograms during theta oscillations demonstrate intermittent trilaminar cell activity (Fig. 8a, d; Table 5). Unlike the majority of hippocampal GABAergic neurons, the trilaminar cell did not fire action potentials on every theta cycle (*n* = 21.8% of all cycles) and when it was active it discharged very few action potentials per cycle. The discontinuous spiking during theta was most pronounced during REM sleep. Interestingly, we also observed a secondary peak in the REM-related autocorrelogram demonstrating an additional slow (1.7 Hz) modulation of the trilaminar cell specifically during this stage of sleep (Fig. 8d). Additionally, in the spike bursts during theta epochs we found three or more action potentials with ISIs shorter than or equal to 12 ms during 6.2% of theta cycles. This is similar to the theta-bursting of three SST+ long-range projecting neurons.

## Discussion

We have analysed the synaptic connectivity and drug-free activity of trilaminar cells (Ferraguti et al. 2005; Sik et al. 1995), one of at least four types of long-range GABAergic projection neurons which are ideally located to promote temporal coordination between the hippocampus and the subiculum (Christenson Wick et al. 2019; Ferraguti et al. 2005; Francavilla et al. 2018; Fuentealba et al. 2008; Harris et al. 2018; Jinno et al. 2007; Sik et al. 1995). We showed that GABAergic neurons with the molecular features of trilaminar cells are embedded into the hippocampal CA1 and CA3 neuronal network, in both the rat and the mouse, with their activity segregated by behavioural and network states. We established that this activity is regulated by inhibitory inputs, including those from the medial septum, which are susceptible to hetero-synaptic modulation of their neurotransmitter release mediated by group III mGluR8a and mGluR7a receptors. Having confirmed certain bias of trilaminar cells towards innervating interneurons in the local CA1 circuit, we suggest that their very fast bouts of activity during slow wave sleep support glutamatergic principal cell assemblies by disinhibition via the transient inhibition of local interneurons.

### Molecular distinction of trilaminar cells and their mGluR8a+ synaptic afferents

We identified trilaminar cells molecularly as SST immunonegative GABAergic neurons with strong somato-dendritic membrane expression of M2 muscarinic acetylcholine receptors and dense innervation by inputs enriched in mGluR8a metabotropic glutamate receptors (see also Ferraguti et al. 2005). In the hippocampus, mGluR8a has been localised to the presynaptic active zone of both glutamatergic and GABAergic terminals forming type I (often called asymmetric) and type II (often called symmetric) synapses, respectively (Ferraguti et al. 2005; Shigemoto et al. 1997), as well as, in cholinergic terminals (Ferraguti and Shigemoto 2006). We have characterised the M2+ postsynaptic neurons and we investigated the neurotransmitter phenotype and the origin of the mGluR8a+ presynaptic terminals.

#### Somato-dendritic M2 expression and acetylcholine

High levels of the inhibitory M2 receptors in the somato-dendritic membranes of trilaminar cells are indicative of their regulation by acetylcholine (Dutar and Nicoll 1988; Seeger and Alzheimer 2001; Tateyama and Kubo 2018; Zheng et al. 2011), but we have not tested any such effects directly. Some interneurons with horizontal dendrites are hyperpolarised by muscarine in stratum oriens (Parra et al. 1998). Furthermore, the non-selective cholinergic agonist carbachol reduces responses to applied glutamate on interneurons in stratum oriens via G-protein activated inwardly rectifying potassium channels, and this modulation is dependent on the expression of M2 (Zheng et al. 2011). Such an inhibitory mechanism is consistent with the low levels of activity of the trilaminar cell we have recorded during theta oscillations associated with exploration or REM sleep, when the cholinergic tone is high in the hippocampus (Kametani and Kawamura 1990; Marrosu et al. 1995). We predict that the cholinergic inhibition of the long-range projecting trilaminar cells contributes to the enhancement of hippocampo-subicular interactions in a network state-dependent manner.

#### GABAergic mGluR8a+ synaptic afferents from the medial septum

We have established that the majority of the mGluR8a+ terminals on M2+ trilaminar cells are GABAergic and that many originate from the medial septum. Indeed, mRNA for mGluR8a has been shown in a subpopulation of medial septal cells (data from the Allen Brain Atlas) although it remains unknown if any of these neurons would project to the hippocampus.

The medial septal terminals presynaptic to trilaminar cells probably originate from at least two still unrecognised different cell types: (1) because of the lack of mGluR8a in medial septal terminals labelled in PV^Cre^ mice and innervating trilaminar cells, we conclude that the mGluR8a+ GABAergic medial septal input identified in VGAT^Cre^ mice originates from PV− cells. Such terminals are similar to those of low-rhythmically firing medial septal cells, which are also PV− (Salib et al. 2019). But whether such cells innervate M2+ neurons in the hippocampus remains to be tested. (2) The numerous PV+ presynaptic terminals making synapses on trilaminar cells would predict a subpopulation of high-rhythmically firing PV+ medial septal cells although these have not been observed to target M2+ hippocampal neurons (Borhegyi et al. 2004; Hangya et al. 2009; Joshi et al. 2017).

#### GABAergic mGluR8a+ synaptic afferents from local interneurons

Local interneurons in the hippocampus are another likely source of the GABAergic and mGluR8a+ terminals innervating trilaminar cells. This is supported by the sparse distribution of mGluR8a mRNA in the hippocampus interpreted as restricted expression in some rare neuron type and not in pyramidal cells (Ferraguti et al. 2005; Harris et al. 2018). With their inputs proven to express VIP, trilaminar cells are likely candidates for receiving inputs from interneuron selective IS-III cells (Acsády et al. 1996; Chamberland et al. 2010; Lacaille et al. 1987; Tyan et al. 2014) immunopositive for both VIP and CR, or from other CR immunonegative VIP+ neurons including VIP-LRPs co-expressing M2 (Acsády et al. 1996; Francavilla et al. 2018; Gulyás et al. 1996; Hájos et al. 1996; Turi et al. 2019). Boutons of the VIP+ IS-III cells express high levels of mGluR7a (Cauli et al. 2000; Dalezios et al. 2002) and we found a high proportion of mGluR7a co-expression in the mGluR8a+ GABAergic terminals innervating trilaminar cells. We confirmed the co-localisation of mGluR8a and VIP in some (as in Ferraguti et al. 2005), but were unable to test the terminals for all three markers. Whether the VIP+ input originated from any of the IS cells remains to be established. In addition, it remains to be determined whether some of the mGluR7a expression in GABAergic terminals (Kinoshita et al. 1998; Somogyi et al. 2003) originates from PV+ local interneurons (Cauli et al. 2000; Dalezios et al. 2002; Somogyi et al. 2003).

#### Cholinergic mGluR8a+ synaptic afferents

Medial septal cholinergic fibres are a major neuromodulatory subcortical pathway in the hippocampus (Gielow and Zaborszky 2017; Lewis et al. 1967; Nyíri et al. 2005). Basal forebrain cholinergic terminals may also use GABA as transmitter and form synapses with interneuron dendrites in the hippocampal CA1 (Takács et al. 2018). In the rat, some cholinergic terminals were identified co-expressing mGluR8a and targeting M2+ cells (Ferraguti and Shigemoto 2006). In our samples, we confirmed the co-expression of VAchT and mGluR8a. Although very few such terminals were detected on single M2+ neurons, these terminals were also immunopositive for VGAT. The physiological roles of a co-modulation of trilaminar cells by GABA and acetylcholine remain to be established.

#### Glutamatergic mGluR8a+ synaptic afferents

Some mGluR8a+ boutons are non-GABAergic and could be glutamatergic. Terminals of hippocampal CA1 pyramidal cells as examined so far lacked mGluR8a expression (Ferraguti et al. 2005) suggesting that the glutamatergic terminals containing these receptors are likely to originate from extrahippocampal sources. There is no evidence that trilaminar cells receive medial septal glutamatergic inputs (Fuhrmann et al. 2015; Justus et al. 2017; Sotty et al. 2003). Glutamatergic medial septal neurons mostly innervate stratum oriens SST+ neurons (Fuhrmann et al. 2015; Justus et al. 2017). Whether these cells are the only targets and whether these glutamatergic axons co-express mGluR8a needs to be addressed in future experiments. Glutamatergic neurons of the amygdala express mGluR8a (Palazzo et al. 2011) and are another candidate to consider as innervating the hippocampus and trilaminar cells (Pikkarainen et al. 1999), but it is unknown if these projections overlap with the somato-dendritic fields of trilaminar cells. There is a marked expression of mGluR8a in the terminal zones of the lateral perforant path originating from principal cells in the entorhinal cortex (Shigemoto et al. 1997). Furthermore, co-localization of mGluR7a and mGluR8a has been shown in the same glutamatergic terminals (Shigemoto et al. 1997). In our current analysis, some of the mGluR7a+/mGluR8a+ input terminals innervating trilaminar cells were immunonegative for GAD and may be glutamatergic. One group of the entorhinal afferents projects to stratum oriens/alveus and shows a higher preference for innervating GABAergic cells (Takács et al. 2012). Several stratum oriens/alveus SST− interneurons were seen decorated by terminals labelled for mGluR7a and mGluR8a (Ferraguti et al. 2005); in some cases, the labelling for the two receptors appeared co-localised in the same terminal (Ferraguti et al. 2005).

We consider the exclusively mGluR7a+ input terminals innervating trilaminar cells as likely originating from the local axon collaterals of CA1 pyramidal neurons (Bradley et al. 1996; Shigemoto et al. 1996). Recurrent collaterals of CA1 pyramidal cells are a common source of glutamate in stratum oriens but their postsynaptic action is differentiated largely due to the selective enrichment of mGluR7a expression in a target cell-dependent manner, i.e. higher levels are distributed to the terminals that form synapses with SST+ O-LM neurons than to terminals making synapses with pyramidal cells or other types of GABAergic neuron including trilaminar cells (Ali and Thomson 1998; Blasco-Ibáñez and Freund 1995; Losonczy et al. 2002; McBain et al. 1994; Shigemoto et al. 1997, 1996; Stachniak et al. 2019).

#### Modulation of trilaminar cell activity by mGluR8a

Although implicated in the pathophysiology of stress-induced disorders including depression and anxiety (Bahi 2017; Fendt et al. 2010; Niswender and Conn 2010), the precise functional roles of mGluR8a remain to be dissected according to cell types and brain areas where the receptor is expressed (Corti et al. 1998; Duvoisin et al. 1995; Ferraguti 2018; O'Connor et al. 2013; Palazzo et al. 2011; Shigemoto et al. 1997). In glutamatergic boutons, mGluR8a may act as autoreceptor activated by glutamate released from the same terminal (Baskys and Malenka 1991; Desai et al. 1994; Gereau and Conn 1995; Scanziani et al. 1998; Shigemoto et al. 1997; Trombley and Westbrook 1992). The presence of mGluR8a in GABAergic terminals indicates heterosynaptic regulation of GABA release (Desai et al. 1994; Gereau and Conn 1995; Hayashi et al. 1993; Kogo et al. 2004; Morishita et al. 1998; Poncer and Miles 1995; Semyanov and Kullmann 2000).

The presynaptic expression of high-affinity mGluR8a in GABAergic boutons innervating trilaminar cells predicts a role in adjusting the inhibition of these neurons depending on the level of glutamatergic activity in the local network. We propose that the intermittent high population synchrony of pyramidal cell action potentials during SWRs results in synaptically released glutamate spill-over (Congar et al. 1997; Scanziani et al. 1997) and suppression of GABA release to trilaminar cells (Capogna 2004; Dammann et al. 2018; Mitchell and Silver 2000; Scanziani et al. 1998; Semyanov and Kullmann 2000; Takahashi et al. 1996; Woodhall et al. 2001), thereby facilitating their prolonged high-frequency burst firing. This allows trilaminar cells to coordinate principal cell activity between the hippocampus and the subiculum during offline processing.

### Output synaptic organisation of trilaminar cells

We have confirmed that trilaminar cells synaptically target both interneurons and to a lesser extent pyramidal cells in the CA1 area, with a bias towards interneurons (Ferraguti et al. 2005). If we consider that the classification criteria of postsynaptic target profiles in the current sample likely leads to an underestimation of targeted interneuron dendritic profiles and a corresponding overestimation of pyramidal cell profiles, the bias for interneurons may be even greater. Around one third of the targets identified as interneurons were PV+ in the rat, and as trilaminar cells formed type II synapses nearly exclusively with dendritic profiles, this makes basket cells, bistratified cells, axo-axonic cells and O-LM cells all candidates for receiving trilaminar cell input (Somogyi and Klausberger 2018). It remains to be determined if trilaminar cells target all types of PV+ cell uniformly or if they have a specific postsynaptic cell type preference. In any case, the preference of trilaminar cells to innervate other GABAergic interneurons points to a more prominent role in the disinhibition of pyramidal cells compared to a minimal contribution to gating of dendritic electrogenesis and burst firing in these same cells (Lovett-Barron et al. 2012; Royer et al. 2012).

In some cases, the synaptic specialisation of trilaminar cell boutons included a row of electron opaque, discrete, spherical bodies in the postsynaptic neuronal profiles aligned with but not attached to the postsynaptic densities. Such rows of synaptic bodies were first observed in the frog ganglion (Taxi 1961), hence named Taxi bodies, and were later described also in the central nervous system mostly in association with type I presumably glutamatergic synapses (Akert et al. 1967; Milhaud and Pappas 1966; Taxi and Babmindra 1972). It is very unusual to find this subsynaptic apparatus in GABAergic synapses, and their presence in trilaminar cell boutons suggests a specialised signalling mechanism with at least some of their postsynaptic neurons.

Due to suboptimal labelling of the distal trilaminar cell terminals in the subiculum, the analysis of the laminar distribution and the target cell types innervated by those boutons could not be performed.

### Comparison of subiculum-innervating trilaminar cells and VIP-LRPs

With the advent of genetic labelling techniques, there has been an expansion in the molecular and functional identification of long-range projecting GABAergic neurons in the hippocampus (Blasco-Ibáñez et al. 1998; Bonifazi et al. 2009; Ceranik et al. 1997; Christenson Wick et al. 2019; Eyre and Bartos, 2019; Francavilla et al. 2018; Fuentealba et al. 2008; Harris et al. 2018; Jinno and Kosaka 2002; Katona et al. 2017; Luo et al. 2019; Melzer et al. 2012; Miyashita and Rockland 2007; Picardo et al. 2011; Yamawaki et al. 2019).

Amongst, the neurons described as long-range projecting and GABAergic, the strongest resemblance to trilaminar cells are the M2+ subgroup of VIP-LRP cells (Type I) of CGE origin identified in the mouse (Francavilla et al. 2018; Luo et al. 2019). These neurons, like the trilaminar cells, are SST immunonegative with their somata and horizontally oriented dendritic trees at the border between stratum oriens/alveus and receive inputs from mGluR8a+ terminals. The local axon collaterals of these cells are concentrated within stratum oriens/alveus, with only a small proportion entering strata pyramidale and radiatum. VIP-LRP cells innervate preferentially interneurons, including PV+/SST+ O-LM cells, bistratified cells and some basket cells (Francavilla et al. 2018; Luo et al. 2019) thereby acting via disinhibitory mechanisms. The target cell types of VIP-LRPs in the subiculum are both interneurons and pyramidal cells (Francavilla et al. 2018); although not necessarily from the same pool as the postsynaptic targets of trilaminar cells.

What distinguishes trilaminar cells molecularly from M2+ VIP-LRP cells (Luo et al. 2019) is the lack of expression of VIP, neuropeptide Y and CB in the cell somata and the very high density of their mGluR8a+ inputs, as we quantified here. We found that VIP-LRP cell markers Netrin-G1 and Proenkephalin-A (Luo et al. 2019) are commonly expressed in the majority of M2+ cells in the mouse (data for the latter not shown), but additional molecular analysis may reveal differences between projection cells in the rat and the mouse. We have identified anatomically VIP-LRP cells in the rat, but recordings of trilaminar cells have not been reported in the mouse. However, single cell mRNA sequencing in the mouse hippocampal CA1 has revealed an isolated cluster of CGE-derived neurons in stratum oriens lacking Sst or Pvalb and expressing Chrm2 and Ntng1, a molecular profile similar to that of trilaminar cells (Harris et al. 2018; Qian et al. 2018). The distinction of this group from VIP-LRPs is not yet clear.

Both, trilaminar cells in the rat and VIP-LRPs in the mouse are more active during non-theta states than during movement related theta oscillations. During quiet wakefulness, trilaminar cells fire at twice the rate as during movement. Similarly, most of the Type I VIP-LRP cells also show increase in action potential generated Ca^2+^ transients during quiet wakefulness, as compared to movement (Francavilla et al. 2018). As found here and also reported under urethane anaesthesia (Ferraguti et al. 2005), the theta-related action potentials of trilaminar cells sometimes cluster into complex spike bursts, and their timing is strongly coupled to the ascending slope of theta but do not occur on every theta cycle. In the mouse, VIP-LRPs were reported (Francavilla et al. 2018) as theta-off or anti-theta cells with unknown theta phase tuning. Anti-theta cells were also described in rats (Colom and Bland 1987; Mizumori et al. 1990).

The most significant distinction between the reported activity of the two cell types is that during SWRs. Under urethane anaesthesia, the trilaminar cell in the rat fires bursts of action potentials of > 200 Hz (Ferraguti et al. 2005). In contrast, the Ca^2+^ transients of VIP-LRPs do not increase in head-fixed awake and resting mice during SWRs (Francavilla et al. 2018). We were unable to detect SWRs in the drug-free rat due to the lack of an independent electrode for the recording of LFPs. However, we recorded bursts of trilaminar cell firing of up to 310 Hz during slow wave sleep which occurred with the frequency of SWRs. Also, strong increases in firing during SWRs matching that of the trilaminar cell have been recorded extracellularly for unlabelled single cells in stratum oriens using tetrodes (unpublished observations).

### Functional implications of the behavioural state-specific activity of trilaminar cells

The contribution of trilaminar cells to the local and inter-regional neuronal assembly co-ordination is network state dependent. Our results distinguish between prolonged burst firing of > 200 Hz during slow wave sleep and SWRs. This is contrasted with a significant reduction in activity during theta oscillations associated with movement and REM sleep.

#### Slow wave sleep and bursting firing

The repeated high-frequency bursts of firing reported under urethane anaesthesia (Ferraguti et al. 2005; Sik et al. 1995) are a conspicuous feature of the trilaminar cell type that may serve to support CA1 principal cells by disinhibition via transient inhibition of certain types of local interneuron. We suggest that this disinhibition facilitates the activation of pyramidal cells into sequences of cell assemblies replayed during SWRs (Diba and Buzsaki 2007; Foster and Wilson 2006). Alternatively, trilaminar cells may entrain specialised GABAergic interneuron types to ripple frequencies which in turn are responsible for the ripple-rhythmic (120–230 Hz) entrainment of pyramidal cells (Csicsvari et al. 1999; Katona et al. 2014, 2017; Lapray et al. 2012; Varga et al. 2012, 2014) thereby initiating their functional coupling by synaptic plasticity mechanisms (Behr et al. 2009; Buzsáki 1989; Commins et al. 1998; Csicsvari et al. 1999; Kokaia 2000; Wilson and McNaughton 1994). An outstanding question remains whether trilaminar cells via their long-range GABAergic axons also transiently inhibit interneurons in the subiculum and therefore facilitate the co-activation of hippocampal and subicular pyramidal cells promoting the oscillatory coherence between these two brain regions (Böhm et al. 2015; Buzsáki 1986, 1989; Chrobak and Buzsaki 1994; Chrobak and Buzsáki 1996; Eller et al. 2015; Norimoto et al. 2013).

#### Reduced activity during theta oscillations

Long-range GABAergic, cholinergic, and glutamatergic projections from the medial septum and diagonal band nuclei (Borhegyi et al. 2004; Colom et al. 2005; Freund and Antal 1988; Hajszan et al. 2004; Joshi et al. 2017; Kimura et al. 1980; Kunitake et al. 2004; Petsche et al. 1962; Unal et al. 2018, 2015; Viney et al. 2018) contribute to the rhythmic entrainment of hippocampal and subicular neurons and the generation of cortical theta oscillations (Brucke et al. 1959; Ford et al. 1989; Hangya et al. 2009; Simon et al. 2006; Sweeney et al. 1992; Varga et al. 2008; Vertes and Kocsis 1997; Wang 2002). Medial septal GABAergic cells have been shown to target exclusively interneurons in the hippocampus (Freund and Antal 1988; Freund and Buzsáki 1996; Joshi et al. 2017; Köhler et al. 1984; Salib et al. 2019; Unal et al. 2018, 2015). We found that trilaminar cells are one of the densely innervated cell types by the GABAergic component of the medial septum, but the theta-related suppression of their activity is markedly different from that of many other identified hippocampal GABAergic neuron type (Joshi et al. 2017; Somogyi et al. 2014). An increase in cholinergic tone, which accompanies theta oscillations, is very likely to contribute to their strong inhibition via M2 receptors (Kametani and Kawamura 1990; Marrosu et al. 1995), as well as, GABAergic gating by VIP+ interneurons. The remarkable decrease in the trilaminar cell firing during theta oscillations may relieve their postsynaptic target interneurons from inhibition. As a result, these interneurons, can orchestrate the theta phase-dependent recruitment of pyramidal cells into temporally ordered local and cross-regional assemblies (Buzsáki 1989, 2010; Commins et al. 1999; Harris et al. 2003; Huang and Kandel 2005; Kokaia 2000; O'Mara et al. 2000; Pastalkova et al. 2008; Somogyi 2010; Wilson and McNaughton 1993, 1994).

## Electronic supplementary material

Below is the link to the electronic supplementary material.

Supplementary file1 (MP4 59199 kb)
